# Two–layer model via non–quasi–periodic normal form theory

**DOI:** 10.1007/s10569-026-10291-5

**Published:** 2026-04-15

**Authors:** Gabriella Pinzari, Benedetto Scoppola, Matteo Veglianti

**Affiliations:** 1 Dipartimento di Matematica “Tullio Levi–Civita”, Università degli Studi di Padova, Via Trieste, 63, 35131 Padova, Italy; 2 Dipartimento di Matematica, Università di Roma “Tor Vergata”, Via della Ricerca Scientifica - 00133, Roma, Italy

**Keywords:** Capture into resonance, Non–quasi–periodic normal form theory, Friction, 37J40, 70F40

## Abstract

The “two–layer model” is a $2+\frac{1}{2}$ degrees–of–freedom non–autonomous dynamical system consisting of a massive, ellipsoidal (possibly spheric) body made of two layers – a hard core and a viscous fluid – revolving about a major planet or a star. We assume that the rotation and the two revolution periods (of core and shell) are close to a resonance, and aim to investigate, in a rigorous way, the mathematical conditions which maintain the resonant motion. In a previous article (Pinzari et al. in Celest Mech Dyn Astron 136(5):39, 2024), we discussed the phenomenon known as “capture into resonance”, via qualitative arguments supported by numerical findings. In this paper, we reframe the model along the lines of a suitable version of (which we refer to as “non–quasi–periodic”) normal form theory and provide an explicit amount of the resonance trapping time, which is estimated as exponentially–long, in terms of the small parameters of the system.

## Introduction

In a previous paper, Pinzari et al. (2024) (in turn based on Goldreich and Peale (1966)), we proposed a model (“two–layer model”, in what follows) for the capture into spin-orbit resonance in which the body is composed by two layer, a lighter shell and an heavier core, interacting via a liquid, or viscous, friction. The model is motivated by the study of the capture into 3:2 resonance of Mercury, in which the viscous friction can be related to a melted mantle between a solid crust and a solid kernel, and by the icy Jupiter’s satellites, that will be studied in a near future by the JUICE mission (Van Hoolst et al. 2024), that are supposed to have a water ocean between the solid icy crust and a rocky core. The model includes a simple — albeit natural — description of the different friction felt by the crust and the core, which is taken proportional to relative velocity (for a simplified model see (Scoppola et al. 2022), other kinds of friction are considered in Rochester (1970); Lhotka et al. (2025)).

In Pinzari et al. (2024), we focused on the study of the equations of motions of the system (see eq. (25) of that paper) resulting from the lower nonzero terms of a time–averaged series expansion of the potential in terms of quite natural small parameters of the system: the eccentricity of the orbit, the a-sphericity of the body and the inverse distance from the sun. Notwithstanding the tailored approximations, the motion equations which we obtained are still non–trivial, due to nonlinearities. Quite surprisingly, based essentially on numerics, we found that such simplified equations provide an account of a possible mechanism of capture into resonance.

The purpose of this paper is to understand under which respect the neglected higher order terms do not interfere with such description. We remark that, by the occurrence of friction, the model is far from being Hamiltonian, whence powerful tools from perturbation theory (like Kolmogorov–Arnold–Moser or Nekhorossev; see below) are not available.

Among the recent theories which deal with friction, conformally symplectic theory is worth to be mentioned (Wojtkowski and Liverani 1998; Calleja et al. 2013; Marò and Sorrentino 2017).

The approach we follow is, in a sense, traditional, in two respects. On the one side, as in Pinzari et al. (2024), we start with a Lagrangian analysis, as we cannot do differently, due to the occurrence of friction. On the other side, we develop a new analytical tool, which we refer to as non–quasi–periodic (NQP, hereafter) normal form theory. In the 70 s of previous Century developing ideas going back to Littlewood, H. Poincaré, Moser, Kolmogorov and his mentor (Hardy et al. 1934; Poincaré 1892; Moser 1961; Birkhoff 1927; Kolmogorov 1954; Arnold 1963b, c), a young student of V.I.Arnold, named Nehorošev (1977, 1979) considered the problem of maximum variation of the action coordinates, *I*, under the action of an Hamiltonian

$$\begin{aligned} H(I, \varphi )=h(I)+\epsilon f(I, \varphi )\qquad 0<\epsilon \ll 1 \end{aligned}$$

which is a slight perturbation of an integrable one, here named as *h*(*I*). Hamiltonians of this kind are common in the literature (e.g., the Hamiltonian of the *n*–body problem, the Euler top, anharmonic interacting oscillators, etc). They are widely studied, since the discovery of the so–called Kolmogorov–Arnold–Moser (KAM) theory (Kolmogorov 1954; Moser 1962; Arnold 1963a), which originated a flow of research still not exhausted, which spreads to dissipative and infinite–dimension systems: see, e.g., Wayne (1990); Kappeler et al. (n.d.); Kuksin and Maiocchi (2018); Sevryuk (2003); Calleja et al. (2017); Berti et al. (2021); Chierchia and Procesi (2022); Calleja et al. (2024) and references therein, for an overview. Nekhorossev proved that, along the motions of *H* the $j^{\textrm{th}}$ action coordinate $I_j$ satisfy an inequality like

$$\begin{aligned} |I_j(t)-I_j(0)|\le \epsilon ^b\qquad \textrm{for}\quad |t|\le \frac{1}{\epsilon }\exp {\left( \frac{1}{\epsilon ^a}\right) } \end{aligned}$$

for suitable positive numbers *a*, *b*. Nekhorossev’s papers had a deep impact on the scientific community. After him, many authors thoroughly studied and progressively clarified the analytic set–up, both in the original setting (Benettin and Gallavotti 1986; Pöschel 1993; Lochak 1992; Lochak and Neĭshtadt 1992; Bounemoura and Niederman 2012; Guzzo et al. 2016) or for systems exhibiting an elliptic equilibrium (Fassò et al. 1998; Guzzo et al. 1998; Pöschel 1999; Pinzari 2013; Bounemoura et al. 2020), or, finally, for numerical approaches (Celletti and Ferrara 1996; Morbidelli and Giorgilli 1997; Froeschlé et al. 2000; Sansottera et al. 2013). An extension of normal form theory to non–autonomous Hamiltonian systems with a special decay of the remainder term *f* has been recently obtained in Fortunati and Wiggins (2016). Recall also Fassò (1989) and references therein for an application to non–hamiltonian vectorfields based on Lie series. Notwithstanding the variety of the recalled analytic results, the occurrence of friction makes them of no practical use to the two–layer model. Using the machinery from Pöschel (1993), we develop a theory for ODEs equipped with vector–fields where, in the lowest approximation, part (possibly, none) of the variables has a quasi–periodic motion, while the other part (possibly, all of them) affords dumped oscillations, i.e., oscillations with complex frequencies, whose real part is negative (even though the theory is meaningful for any complex value of the frequency). Previous similar statements appeared in the unpublished note^1^ and, later, in Chen and Pinzari (2021); see (Pinzari 2023) for a review.

Apart from its interest from a technical point of view, we believe that our result is physically meaningful, because it allows, quite constructively (meaning that all the constants involved may be estimated through the proofs: see, e.g., Eq.s (15), (18)), to ensure that the motions of the relevant quantities in the two–layer model are close to such dumped oscillations, for exponentially–long times.

In particular, we are able to exhibit an explicit value of the time *T* such that for $t<T$ the solution of the linear system stays close, in a suitable norm, to a dumped oscillation, and to compare it with the characteristic time $\tau$ ruling the exponential decay, given by the inverse of the modulus of the real part of the frequencies. As a matter of fact, if $T>\tau$, the solution will never escape from the equilibrium, at all times. We remark at this respect that the specificities of the problem at hand allow us to reformulate the underlying, rather complicated, fourth–order eigenvalue equation as second–order ODE and to treat it via the min–max principle eventually (see also Remark 5.1 below).

This paper is organized as follows. In the next Sect. 2, we recall the basic framework of Pinzari et al. (2024), so as to derive the explicit form of the motion equations (2), and state our main result, Theorem 2.1. In Sect. 3 we state precisely the aforementioned NQP normal form theory (see Theorems 3.1, 3.2) and prove Theorem 2.1 and, in Sects. 4.1 and 4.2 we prove Theorems 3.1, 3.2, respectively. In Sect. 5, we provide the mentioned upper and lower bounds of the size of dumping. Finally, we dedicate Appendix A to recall an abstract result on actions of change of coordinates to vector–fields, which may turn to be useful to non–expert readers.

## Lagrangian set–up and result

### Description of the model

It is usually believed (and partly proved) that, in general, resonances carry instability in conservative, close–to–be–integrable systems, up to the point of causing ejections of planets or collisions. Still, resonances are ubiquitous in planetary models and look stable over million of years. The idea underlying this section (or, more in general, this note) is to show a mechanism which stabilizes resonances through friction. In particular, we focus on a variation of the well known spin–orbit problem, which we name, as in the title, *two–layer model*. This is a $2+{\frac{1}{2}}$ degrees–of–freedom dynamical system, constructed as follows. With reference to (Pinzari et al., 2024, Fig. 1), we consider an extended body with total mass *m* (denoted as *P*, “planet”, in what follows) moving on a plane and undergoing gravity attraction by a point-wise attracting mass *M* (*S*, “sun”). For simplicity, we assume that *S* is fixed in some point of the plane and that the center of *P* describes a Keplerian, elliptic orbit ${\mathcal {E}}$, with one of its foci at *S* and fixed semi–major axis *a*, perihelion direction ${{{\textbf {i}}}}$ and eccentricity *e* (we assume the perihelion is well defined, namely, $e\ne 0$). The position of *P* on ${\mathcal {E}}$ is determined by the value of the “mean anomaly” $\ell$, which evolves linearly in time, accordingly to Kepler law:

1 $$\begin{aligned} {\dot{\ell }}=\omega \qquad \omega :=2\pi \sqrt{\frac{GM}{a^3}} \end{aligned}$$

with *G* the gravity constant. Concerning the shape and structure of *P*, we assume it consists of two thickless layers (called “core” and “shell” in what follows), both having elliptic shape, but possibly oriented in different directions. The different orientation of the two ellipses is physically interpreted as evidence of mutual friction between the layers, which, as well as in Pinzari et al. (2024), we aim to take into proper consideration. In addition, for the core and the shell we consider only motions which are close to be “resonant” (namely, with periods ratios close to a rational number) with the revolutions of *P* about *S*. As, due to the friction, the energy is not conserved, the Hamiltonian analysis (and its powerful machinery) is not an option. We then proceed with a Lagrangian analysis, as this allows to set friction forces in via a Reileigh function *R*. To choose the Lagrangian coordinates, we fix a reference frame with the first axis in the direction ${{{\textbf {i}}}}$ of the perihelion of ${\mathcal {E}}$. We denote as $\rho =\rho (a, e, \ell )$ the position of the center of *P* relatively to *S*; as $\varphi$ and $\nu$ the angles formed by $\rho$ and the semi–major axes directions of the shell and the core. Since our purpose is to study motions for $\varphi$ and $\nu$ which are close to a $2(k/2 +1): 2(k/2 +1): 2$ ratio between $\varphi$, $\nu$ and $\ell$ (“spin–orbit resonance”), we introduce the quantities $\gamma$ and $\eta$ via

$$\begin{aligned} \varphi = \frac{k \ell }{2} + \gamma \text { and } \nu = \frac{k \ell }{2} + \eta . \end{aligned}$$

The motion of $\gamma$ and $\eta$ is determined by the two second–order equations

2 $$\begin{aligned} \left\{ \begin{array}{lll} \frac{d}{dt} \left( \frac{\partial \mathcal {L}}{\partial {\dot{\gamma }}} \right) = \frac{\partial \mathcal {L}}{\partial \gamma } + \frac{\partial R}{\partial {\dot{\gamma }}}\\ \frac{d}{dt} \left( \frac{\partial \mathcal {L}}{\partial {\dot{\eta }}} \right) = \frac{\partial \mathcal {L}}{\partial \eta } + \frac{\partial R}{\partial {\dot{\eta }}}. \end{array} \right. \end{aligned}$$

The explicit expressions of ${{\mathcal {L}}}$ and *R* are (compare with equations (Pinzari et al., 2024, (15), (16), (23), (24)). Beware that $\beta$ and $\beta '$ correspond to $\lambda$ and $\lambda '$ in Pinzari et al. (2024).)

$$\begin{aligned}{\mathcal {L}}= {\mathcal {L}}_\gamma + {\mathcal {L}}_\eta -\widehat{\mathcal {V}},\qquad R= -\frac{1}{2}\beta \left( \dot{\gamma } - \dot{\eta } \right) ^2 - \frac{1}{2}\beta ' \left( \dot{\eta } + \frac{k\omega }{2}\right) ^2, \end{aligned}$$

with $\beta > 0$ a “viscous friction coefficient”, $\beta ' > 0$ a “viscoelastic friction coefficient’, and

3 $$\begin{aligned} {\mathcal {L}}_\gamma= &  \frac{1}{2} C' \left[ \frac{k}{2}\omega + \dot{\gamma } + {\dot{\vartheta }}(\ell , \omega , e)\right] ^2 + \frac{3}{8}\omega ^2(B'-A') a_k(e)e^k\cos 2\gamma -\widetilde{\mathcal {V}}'(\ell , \gamma , e)\nonumber \\ {\mathcal {L}}_{\eta }= &  \frac{1}{2} C \left[ \frac{k}{2}\omega +{ \dot{\eta }} + {\dot{\vartheta }}(\ell , \omega , e)\right] ^2 + \frac{3}{8}\omega ^2(B-A) a_k(e)e^k\cos 2\eta -\widetilde{\mathcal {V}}(\ell , \eta , e) \nonumber \\ \widetilde{\mathcal {V}}, &  \widetilde{\mathcal {V}}' =\textrm{O}\left( \frac{r^2}{a^3}\right) ,\quad \widehat{\mathcal {V}}=\textrm{O}\left( \frac{r^3}{a^4}\right) \end{aligned}$$

where $\widetilde{\mathcal {V}}(\ell , \eta , e)$, $\widetilde{\mathcal {V}}'(\ell , \eta , e)$ have vanishing^2^$\ell$–average and with *r* being the average radius of *P*. To lighten notations, we introduce the homogeneous quantities

$$\begin{aligned}c_1:= &  \frac{3}{4} \frac{B'-A' }{C'} {\omega ^2} {e^k} a_k(e),\quad \theta :=\frac{\beta }{C'}\nonumber \\ c_2:= &  \frac{3}{4} \frac{B-A }{C}\omega ^2 e^k a_k(e),\quad \epsilon :=\frac{\beta }{C},\quad \upupsilon :=\frac{\beta '}{C},\quad -v_0:=\frac{k}{2} \nonumber \\ {\widetilde{P}}_\gamma:= &  -\frac{\partial _\gamma \widetilde{\mathcal{V}}'}{C'},\quad \widehat{P}_\gamma :=-\frac{\partial _\gamma \widehat{{\mathcal {V}}}'}{C'},\quad {\widetilde{P}}_\eta :=-\frac{\partial _\eta \widetilde{\mathcal{V}}}{C},\quad {\widehat{P}}_\eta :=-\frac{\partial _\eta \widehat{\mathcal{V}}}{C} \end{aligned}$$

Here, *A*, *B*, *C*, $A'$, $B'$, $C'$ are the moments of inertia of core and shell, respectively; *e*, *a* are eccentricity and semi–major axis of the ellipse, $\omega$ is the Keplerian frequency given in (1). We recall that the functions $a_k(e)$ are^3^ power series of *e* starting with 1. The coefficients $c_1$, $c_2$ are related to the geometry and the order of resonance, while $\theta$, $\epsilon$ and $\upupsilon$ are related to the friction coefficients $\beta$, $\beta '$. From the physical meaning of such coefficients, in this paper we shall always regard

5 $$\begin{aligned} \theta>\epsilon >\upupsilon \end{aligned}$$

even though more precise quantitative relations will be specified.

With the above definitions and notations, we rewrite Equations (1) and (2) in the form of first order ODEs system

6 $$\begin{aligned} \left\{ \begin{array}{llll} {\dot{\gamma }}= p_\gamma \\ \dot{p}_\gamma = - c_1 \sin {2 \gamma } - \theta (p_\gamma - p_\eta )+{\widetilde{P}}_\gamma +{\widehat{P}}_\gamma \\ {\dot{\eta }}=p_\eta \\ \dot{p}_\eta = - c_2 \sin {2\eta } + \epsilon (p_\gamma - p_\eta ) -\upupsilon (p_\eta - v_0)+{\widetilde{P}}_\eta +{\widehat{P}}_\eta \\ {\dot{\ell }}=\omega \end{array}\right. \end{aligned}$$

Neglecting the *P*’s releases^4^ the system considered in Pinzari et al. (2024). The system (6) will be referred to as *full system* in what follows. Such locution is to be understood as opposite to *linearized system*, which is a further simplification, not considered in Pinzari et al. (2024), and now we describe.

### The linearized system and result

We consider the point $(0, 0, \eta _0, 0)$, where

$$\begin{aligned} \eta _0:=\frac{1}{2}\sin ^{-1}\left( \frac{\upupsilon }{c_2}v_0\right) \quad \mod \pi . \end{aligned}$$

The point $(0, 0, \eta _0, 0)$ is an equilibrium of the vector–field obtained from the first four equations in (6) by neglecting all the *P*’s. It is well–defined provided that

$$\begin{aligned}\frac{\upupsilon }{c_2}|v_0| <1.\end{aligned}$$

An expansion about such equilibrium leads to the system

7 $$\begin{aligned} \left\{ \begin{array}{llll} {\dot{\gamma }}=p_\gamma \\ \dot{p}_\gamma = - 2c_1\, \gamma - \theta p_\gamma +\theta p_{\psi } +\breve{P}_\gamma +\widetilde{P}_\gamma +{\widehat{P}}_\gamma \\ {\dot{\psi }}=p_{\psi }\\ \dot{p}_{\psi } = - 2\bar{c}_2 \,\psi + \epsilon p_\gamma -\delta p_{\psi } +\breve{P}_u+{\widetilde{P}}_u+{\widehat{P}}_u\\ {\dot{\ell }}=\omega \end{array}\right. \end{aligned}$$

where $\eta$ has been changed with $\psi :=\eta -\eta _0$,

$$\begin{aligned}\delta :=\epsilon +\upupsilon ,\ \bar{c}_2:=c_2\cos 2\eta _0 \end{aligned}$$

${\widetilde{P}}_\gamma$, ${\widehat{P}}_\gamma$, ${\widetilde{P}}_u$, ${\widehat{P}}_u$ denoting (with abuse) the previous functions in the new variables, and

8 $$\begin{aligned} \breve{P}_\gamma (\gamma , \psi , \ell ):=- c_1 \sin 2\gamma +2c_1\gamma ,\quad \breve{P}_u(\gamma , \psi , \ell ):= - c_2\sin {2(\psi +\eta _0)}+ \upupsilon v_0+ 2\bar{c}_2\,\psi \end{aligned}$$

being the higher order terms released from the expansion. From now on, we shall neglect to write the “bar” in (7). Neglecting all the *P*’s the system decouples as a linear one involving the “slow” variables $(\gamma , p_\gamma , \psi , p_{\psi })$, and hence named *linearized system*,

9 $$\begin{aligned} \left\{ \begin{array}{llll} {\dot{\gamma }}=p_\gamma \\ \dot{p}_\gamma = - 2c_1\, \gamma - \theta p_\gamma +\theta p_{\psi } \\ {\dot{\psi }}=p_{\psi }\\ \dot{p}_{\psi } = - 2c_2 \,\psi + \epsilon p_\gamma -\delta p_{\psi } \end{array}\right. \end{aligned}$$

plus the equation (1) for the “fast” variable $\ell$, left apart. We denote as *L* the matrix of the coefficients of (9). We have

#### Proposition 2.1

For values of $c_1$, $c_2$, $\theta$, $\epsilon$ and $\delta$ such that the resolvent of the characteristic polynomial of *L* does not vanish, and if the inequality in (5) and

10 $$\begin{aligned} \epsilon \ne 0,\quad \theta ^2<\frac{4}{9}\min \{c_1,c_2\} \end{aligned}$$

hold, then *L* admits two distinct complex–conjugated couples of eigenvalues

11 $$\begin{aligned} \lambda _1=a_1+\textrm{i} \Omega _1,\quad \lambda _2=a_2+\textrm{i} \Omega _2,\quad \lambda _3=a_1-\textrm{i} \Omega _1,\quad \lambda _4=a_2-\textrm{i} \Omega _2 \end{aligned}$$

with strictly negative real parts $a_i$ and non–vanishing imaginary parts $\Omega _i$. More precisely, the following bound holds

12 $$\begin{aligned} {\, \mathrm Re\, }\lambda _j\subset \left[ -\frac{3}{2}\theta ,-\frac{\upupsilon }{{6}}\right] ,\quad {|{\, \mathrm Im\, }\lambda _j|\subset \left[ \frac{\epsilon }{\theta }\sqrt{\min \{c_1, c_2\}},\ \frac{\theta }{\epsilon }\sqrt{2\max \{c_1, c_2\}}\right] }. \end{aligned}$$

The proof of Proposition 2.1 is provided in Sect. 5.

Proposition 2.1 implies that the motions of $\gamma$ and $\psi$ along the solutions of the linearized system are given by

13 $$\begin{aligned} \gamma _*(t):=\sum _{j=1}^4b_{1j} e^{\lambda _j t},\qquad \psi _*(t):=\sum _{j=1}^4b_{3j} e^{\lambda _j t} \end{aligned}$$

where $b=(b_{ij})$ is such that $b^{-1}L b$ is in diagonal form. Our purpose is to continue the motions (13) to the full system.

We shall be able to prove that such continuation exists under two conditions, which in a sense are rather classical. The former condition is related to the numbers $a_i$, $\Omega _i$ in (11), for which we require that a strong non–resonance holds. The condition we take resembles the classical “Diophantine” condition, and is stated as follows:

14 $$\begin{aligned} &  \forall \ (\alpha , q)\in {\mathbb {N}}\times {\mathbb {Z}}: \quad \alpha -|q|\ge 0,\quad (-1)^{\alpha -|q|}=1 \nonumber \\ &  |a_i\alpha -a_j|\ge \frac{\upupsilon }{(\alpha +1)^\tau }\quad \textrm{or}\quad |\Omega _i q-\Omega _j|\ge \frac{\upupsilon }{(|q|+1)^\tau }\quad \forall i\ne j\in \{1,\ 2\}.\end{aligned}$$

The second assumption is, as it always happens in similar contexts, is a “smallness” condition which ensures that the full system is close enough to the averaged and linearized one given in (9). It is worth remarking, at this respect, that the complexity of the problem is such that there is not a single parameter which makes the remainder small. Rather, the smallness is required by asking that a suitable combination of the quantities involved (masses, distances, moments of inertia ratio, friction coefficients, etc) is small. Compare Equations (15) and (18) below.

#### Theorem 2.1

Under condition (14) and the assumptions of Proposition 2.1, there exist numbers $c>1$ and $\varepsilon _*<1$ such that for any choice of $c_1$, $c_2$, $\epsilon$, $\delta$, $\upupsilon$, $v_0$, *r*, *a*, *C*, $C'$ such that, if the quantities

15 $$\begin{aligned} \mu _0:= &  \max \left\{ c_1,\ c_2,\ \varepsilon _0,\ \epsilon \varepsilon _0,\ \delta \varepsilon _0,\ |v_0|\upupsilon ,\ \frac{r^2}{a^3\varepsilon _0\min \{C,\ C'\}},{\frac{\theta }{\epsilon }max\{c_1, c_2\}} \right\} \nonumber \\ \mu _1:= &  \max \left\{ \frac{\mu _0^2}{\omega },\ \mu _0\frac{r}{a},\ c_1\varepsilon _0^3,\ c_2\varepsilon _0^2,\ {\frac{\epsilon }{\theta }min\{c_1, c_2\}}\right\} \end{aligned}$$

verify

16 $$\begin{aligned} {c}\frac{\mu _0}{\omega }\le 1,\qquad {c}\frac{\mu _1}{\upupsilon }\le 1 \end{aligned}$$

then all the solutions of the system of ODEs (7) with initial datum in $B_{\varepsilon _*}$ verify

17 $$\begin{aligned} |\gamma (t)-{\hat{\gamma }}(t)|< c\,\varepsilon ,\quad |\psi (t)-\hat{\psi }(t)|< c\, \varepsilon \qquad \forall \ |t|< T \end{aligned}$$

where ${\hat{\gamma }}(t)$, $\hat{\psi }(t)$ have the expression in (13), with $\lambda _j$ replaced by suitable ${\hat{\lambda _j}}$ verifying

$$\begin{aligned} {\, \mathrm Re\, }{\hat{\lambda _j}}<0,\quad |{\hat{\lambda _j}}-\lambda _j|\le \frac{\mu _1}{2} \end{aligned}$$

while $\varepsilon$ and *T* are given by

18 $$\begin{aligned} &  \varepsilon :=\max \left\{ \frac{\mu _0}{\omega },\ \left( \frac{\mu _1}{\upupsilon }\right) ^{\frac{1}{\tau +1}}\right\} ,\quad T\ge \frac{1}{c\upupsilon }\varepsilon ^{-\tau }e^{\frac{1}{c\varepsilon }} \end{aligned}$$

where $\tau$ is the number in (14).

#### Remark 2.1

As a comparison with Pinzari et al. (2024), we emphasize that, while the system considered in our previous paper is time–averaged and truncated to the lowest order terms with respect to distances, Theorem 2.1 is tailored on the *full* system, however regarded as a small perturbation of the *linearized* and time–averaged equations. In particular, in the present paper we are far from proving, rigorously, the trapping mechanism naively sketched in (Pinzari et al., 2024, Figure 2). Rather, Theorem 2.1 (namely, Equation (17)) says that the motions of the full system remain $\varepsilon$–close to the solutions (13) of the averaged and linearized system for $\varepsilon ^{-1}$–exponentially–long times, where $\varepsilon$ is defined through (15) and (18).

## Proof of Theorem 2.1 via NQP normal form theory

The proof of Theorem 2.1 uses a formulation of normal form theory for vector–fields, carefully designed around the system (7). More precisely, it is based on two results (Theorem 3.1 and Theorem 3.2 below) which here we quote together with the necessary background of notations and definitions. While the proof of such results is deferred to the next sections, here we prove how Theorem 2.1 follows from them.

### 3.1 Definitions and notations

• We fix a norm $|\cdot |$ in $\mathbb {C}^p$ and then: For a given set $A\subset {\mathbb {R}}^p$ and $r>0$, we let

$$\begin{aligned} A_r:=\bigcup _{x\in A}B_r(x) \end{aligned}$$

where $B_r(x)$ is the complex ball with radius *r* centered at *x*:

$$\begin{aligned} B_r(x):=\big \{z\in {\mathbb {C}}^p:\ |z-x|<r\big \}. \end{aligned}$$

• We denote as ${\mathcal {O}}_{u}$, with $u:=(\varepsilon , s)$, the space of vector–fields

19 $$\begin{aligned} Z=(Z_1,\ldots ,Z_{m+n}):\quad V_u:=B^m_\varepsilon \times {\mathbb {T}}^n_s\rightarrow { \mathbb {C}}^{m+n} \end{aligned}$$

which are holomorphic on $V_{u_0}$, $u_0=(\varepsilon _0, s_0)$, with some $\varepsilon _0>\varepsilon$, $s_0>s$.

• If

$$\begin{aligned}Z_h=\sum _{\alpha , k} z_{\alpha k}^h\zeta ^\alpha e^{\textrm{i}(k\cdot \varphi )}\end{aligned}$$

denotes the Taylor–Fourier expansion, we define the *weighted norms* as

20 $$\begin{aligned} |X|_{u}^{w}:= &  \sum _{h=1}^{m+n} w^{-1}_h|Z_h|_{u},\quad |\!|\!|X |\!|\!|_{u}^{w}:=\sum _{h=1}^{m+n} w^{-1}_h\Vert Z_h\Vert _{u}\end{aligned}$$

where

$$\begin{aligned}|Z_h|_{u}:=\sup _{V_u}|Z_h|,\quad \Vert Z_h\Vert _{u}:= \sum _{\alpha , k} |z_{\alpha k}^h|\varepsilon ^{|\alpha |_1} e^{|k|_1 s}\end{aligned}$$

and with $w=(w_1$, $\ldots$, $w_{m+n})\in {\mathbb {R}}_+^{m+n}$ the *weights*.

• For each $1\le h\le m+n$, let $p_h\in {\mathbb {N}}^{m+n}$ be defined so that

21 $$\begin{aligned} p_h=(p_{h1}, \ldots , p_{h, {m+n}}),\quad p_{hj}=\left\{ \begin{array}{lll} \delta _{hj}\quad & \textrm{if}\ h=1,\ldots ,m\\ 0& \textrm{if}\ h=m+1,\ldots ,m+n \end{array} \right. \end{aligned}$$

where $\delta _{hj}$ is the Kronecker symbol. The quantity

22 $$\begin{aligned} \textrm{d}_{\alpha , k}^h(\lambda , \omega ):=-\Big (\big (\alpha , k\big )-p_h\Big )\cdot (\lambda , \textrm{i}\omega ). \end{aligned}$$

will be referred to as $h^{\textrm{th}}$ *small denominator*.

• If $\gamma \ge 0$, define the $(m+n)$–ple of lattices $\Lambda ^\gamma =(\Lambda ^\gamma _1,\ldots , \Lambda ^\gamma _{m+n})$

23 $$\begin{aligned} \Lambda ^\gamma _h(\lambda , \omega ):=\left\{ (\alpha , k)\in {\mathbb {N}}^m\times {\mathbb {Z}}^n:\ |\textrm{d}_{\alpha , k}^h(\lambda , \omega )|\le \gamma \right\} \end{aligned}$$

If $\gamma =0$, the $(m+n)$–ple $\Lambda ^0=(\Lambda ^0_1,\ldots , \Lambda ^0_{m+n})$ will be also referred to as *exactly resonant lattices* $(m+n)$–*ple*. If $\gamma >0$ they will be called $\gamma$–robust resonant lattices $(m+n)$–ple $\Lambda _h\subset {\mathbb {N}}^m\times {\mathbb {Z}}^n$.

• The projectors $T_K Z$, $\Pi _{{\Lambda }}Z$ will denote the vector–fields defined via

24 $$\begin{aligned} (T_KZ)_h:=\sum _{|(\alpha , k)|< K} z_{\alpha k}^h\zeta ^\alpha e^{\textrm{i}(k\cdot \varphi )}\qquad (\Pi _{{\Lambda }}Z)_h:=\Pi _{{\Lambda }_h}Z_h:=\sum _{(\alpha , k)\in \Lambda _h} z_{\alpha k}^h\zeta ^\alpha e^{\textrm{i}(k\cdot \varphi )}. \end{aligned}$$

where the norm |*q*| of an integer vector $q=(q_1,\ldots ,q_p)$ is $\sum _{i=1}^p |q_i|$.

### Normal form theorems

We consider a system of ODEs

25 $$\begin{aligned} \dot{x}=X(x) \end{aligned}$$

where $x:=(\zeta , \varphi )\in B\times {\mathbb {T}}^n$, with $B\subset {\mathbb {R}}^m$ is a neighborhood of $0=(0, \ldots , 0)$, *X*(*x*) is a vector–field having the form

26 $$\begin{aligned} X(x)=N(x)+ P(x) \end{aligned}$$

where *N*(*x*) is $\varphi$–independent and given by

27 $$\begin{aligned} N(\zeta )=\left( \begin{array}{ccc} \lambda _1 \zeta _1\\ \vdots \\ \lambda _m\zeta _m\\ \omega _1\\ \vdots \\ \omega _n \end{array}\right) \end{aligned}$$

with suitable $\lambda \in {\mathbb {C}}^m$, $\omega \in {\mathbb {C}}^n$. Then we have

#### Theorem 3.1

Let $\gamma >0$, $1\le K\in \mathbb {N}$, $X\in {\mathcal {O}}_{u}$ be as in (26), with *N* as in (27), $u=(\varepsilon , s)$, $w=(\rho ,\sigma )<u/2$. Assume that

28 $$\begin{aligned} e \gamma ^{-1} |\!|\!|P|\!|\!|^{w} _{u}<1 \end{aligned}$$

Then there exists a holomorphic change of coordinates

$$\begin{aligned} \phi _+:x_+=(\zeta _+, \varphi _+)\rightarrow x=(\zeta , \varphi ) \end{aligned}$$

which carries *X* to $X_+\in {\mathcal {O}}_{u-2w}$, with $X_+=N+G_++ P_+$, where

29 $$\begin{aligned} G_+= \Pi _{{\Lambda }^\gamma } T_{2K} P.\end{aligned}$$

Moreover, there exists $Y\in {\mathcal {O}}_{u}$ such that $X_+:=e^{{\mathcal {L}}_Y}X$ and

30 $$\begin{aligned} |\!|\!|P_+|\!|\!|^{w}_{u-2w} \le \frac{1 }{1-e \gamma ^{-1} \left|\!|\!|P\right|\!|\!|^w_{u}}\left( e \gamma ^{-1}|\!|\!|P|\!|\!|^w_{u}|\!|\!|P|\!|\!|^w_{u-w}+e^{-K \tau }\left|\!|\!|P\right|\!|\!|^w_{u}\right) \end{aligned}$$

with $\tau$ as in (76) and $|\!|\!|Y|\!|\!|^w_{u}\le \gamma ^{-1}|\!|\!|P|\!|\!|^w_{u}$. Finally, the transformation $\phi _+$ verifies

31 $$\begin{aligned} |\phi _+-\textrm{id}|_{u-2w}^w\le \gamma ^{-1}|\!|\!|P|\!|\!|_u^w \end{aligned}$$

#### Theorem 3.2

There exists $C_*>0$ such that the following holds. Let $\gamma >0$, $1\le K\in \mathbb {N}$, $X\in {\mathcal {O}}_{u}$ be as in (26), with *N* as in (27), $u=(\varepsilon , s)$, $w=(\rho ,\sigma )<u/4$. Put ${\bar{\sigma }}:=\min \{\sigma , \rho /\varepsilon \}$. Assume that

32 $$\begin{aligned} K{\bar{\sigma }}\ge \log (12) \end{aligned}$$

and that *P* is so small that

33 $$\begin{aligned} C_* K {\bar{\sigma }} \gamma ^{-1} |\!|\!|P|\!|\!|^{w} _{u}<1\qquad \end{aligned}$$

Then there exists a holomorphic transformation of coordinates

$$\begin{aligned} \phi _*:\quad V_{u-4w}\rightarrow V_{u} \end{aligned}$$

which carries *X* to

$$\begin{aligned}X_*=N+G_*+ P_*\in {\mathcal {O}}_{u-4w}\end{aligned}$$

with $$G_*$$ verifying

$$\begin{aligned} G_*={\Pi } _{{\Lambda }^\gamma }T_{2K} G_*,\qquad |\!|\!|G_*-\Pi _{{\Lambda }^\gamma }T_{2K} P|\!|\!|^w_{u-4w}\le 8 e \gamma ^{-1}\left( |\!|\!|P|\!|\!|^w_{u}\right) ^2 \end{aligned}$$

and $$P_*$$ “small”:

$$\begin{aligned} |\!|\!|P_*|\!|\!|^{w}_{u-4w} \le e^{-K {\bar{\sigma }}/4}\left|\!|\!|P\right|\!|\!|^w_{u} \end{aligned}$$

Finally, the transformation $\phi _*$ verifies

34 $$\begin{aligned} |\phi _*-\textrm{id}|_{u-4w}^w\le 2\gamma ^{-1}|\!|\!|P|\!|\!|_{u}^{w}. \end{aligned}$$

#### 3.3 Proof of Theorem 2.1

We consider the sets $\Lambda _h^{\gamma }$ defined in (23), with $m=4$, $n=1$, $h=1$, 2, 3, 4, 5, $\lambda _i$ as in Proposition 2.1 and $\omega$ the Keplerian frequency (1). We write the product of projectors

$$\begin{aligned} \Pi _{\Lambda }T_{2K}=\Pi _{\Lambda ^{\gamma , 2K}} \end{aligned}$$

where $\Lambda _h^{\gamma , 2K}:=\Lambda _h^{\gamma }\cap \{|(\alpha , k)|<2K\}$, and we study the sets $\Lambda _h^{\gamma , 2K}$.

##### Proposition 3.1

Let $p_h$ be as in (21), $h\in \{1$, 2, 3, 4, $5\}$. If inequalities

35 $$\begin{aligned} (2K+1)\frac{\theta }{\epsilon }\sqrt{2\max \{c_1, c_2\}}<\omega ,\quad \gamma <\min \left\{ \frac{\upupsilon }{ 6},\ 2\frac{\epsilon }{\theta }\sqrt{\min \{c_1, c_2\}},\ \frac{\upupsilon }{(2K+1)^{\tau }}\right\} \end{aligned}$$

are satisfied, then $\Lambda _h^{\gamma , 2K}=\{p_h\}$, for all *h*.

##### Proof

We begin with $h=5$. $p_5$ is the null vector (0, 0, 0, 0, 0), hence (see definitions (21) and (22))

$$\begin{aligned} \textrm{d}^5_{\alpha , k}(\lambda , \omega )=-(\lambda \cdot \alpha +\textrm{i} k\omega ). \end{aligned}$$

We prove that

36 $$\begin{aligned} |\textrm{d}^5_{\alpha , k}(\lambda , \omega )|\ge \frac{\upupsilon }{ 6}\quad \forall \ (\alpha , k)\in {\mathbb {N}}^5\times {\mathbb {Z}}\setminus \{(0,0,0,0,0)\}. \end{aligned}$$

Let $(\alpha _1, \alpha _2, \alpha _3, \alpha _4, k)\in {\mathbb {N}}^4\times {{\mathbb {Z}}}{\setminus } \{(0,0,0,0)\}$. If $(\alpha _1, \alpha _2, \alpha _3, \alpha _4)\ne (0,0,0,0)$, then, as $\alpha _j\ge 0$ and ${\, \mathrm Re\, }\lambda _j<0$ for all $1\le j\le 4$, by (12),

$$\begin{aligned} |\textrm{d}^5_{\alpha , k}(\lambda , \omega )|\ge &  \left| \sum _{j=1}^4\alpha _j {\, \mathrm Re\, }\lambda _j\right| =\sum _{j=1}^4\alpha _j|{\, \mathrm Re\, }\lambda _j|\ge \min _{1\le j\le 4}|{\, \mathrm Re\, }\lambda _j|\ge \frac{\upupsilon }{ 6}. \end{aligned}$$

If, on the other hand, if $(\alpha _1, \alpha _2, \alpha _3, \alpha _4)=(0,0,0,0)$ and $k\ne 0$, one has

$$\begin{aligned}|\alpha \cdot \lambda +\textrm{i}\omega k|=\omega |k|\ge \omega \ge \frac{\upupsilon }{ 6}.\end{aligned}$$

Then (36) is completely proved.

Now we consider the case $h=1$. $p_1$ is the vector (1, 0, 0, 0, 0), hence,

37 $$\begin{aligned} \textrm{d}^1_{\alpha , k}(\lambda , \omega )=-(\lambda \cdot \alpha -\lambda _1+\textrm{i} k\omega ). \end{aligned}$$

We prove that

38 $$\begin{aligned} |\textrm{d}^1_{\alpha , k}(\lambda , \omega )|\ge \min \left\{ \frac{\upupsilon }{ 6},\ 2|\Omega _1|,\ \frac{\upupsilon }{(2K+1)^{\tau }} \right\} \quad \forall \ (\alpha , k)\in {\mathbb {N}}^3\times {\mathbb {Z}}\setminus \{(1,0,0,0,0)\}. \end{aligned}$$

We distinguish three cases, according to the values of $(\alpha , k)$, which corresponds to subdivide $\Lambda _1^{\gamma , K}$ into proper regions.

*Case* $\alpha _1\ge 1$, $(\alpha , k)\ne (1,0,0,0,0)$. Proceeding as in the previous case (but with $\alpha _1$ replaced by $\alpha _1-1\ge 0$), we equally conclude

39 $$\begin{aligned} |\textrm{d}^1_{\alpha , k}(\lambda , \omega )|\ge \frac{\upupsilon }{ 6}\quad \forall \ (\alpha , k)\in {\mathbb {N}}^5\times {\mathbb {Z}}\setminus \{p_1\},\ \alpha _1\ge 1. \end{aligned}$$

*Case* $\alpha _1=0$, $\alpha _3\ne 0$. Using (11) into (37), we get

40 $$\begin{aligned} |\textrm{d}^1_{\alpha , k}(\lambda , \omega )|= &  \Big | (\alpha _2+\alpha _4) a_2+(\alpha _3-1)a_1 +\textrm{i}\Big ( -(\alpha _3+1)\Omega _1+(\alpha _2-\alpha _4)\Omega _2 +k\omega \Big )\Big |\nonumber \\ &  \textrm{if}\ \alpha _1=0\end{aligned}$$

Then

41 $$\begin{aligned} |\textrm{d}^1_{\alpha , k}(\lambda , \omega )|\ge \left\{ \begin{array}{lll} \min _i|\Re \lambda _i|\ge \frac{\upupsilon }{6}\quad & \textrm{if}\ (\alpha _2, \alpha _3, \alpha _4)\ne (0,1,0)\\ \\ \omega -2|\Omega _1|\ge \frac{\omega }{2}& \textrm{if}\ (\alpha _2, \alpha _3, \alpha _4)= (0,1,0)\,\ k\ne 0\\ \\ 2\inf |\Omega _1|\ge \frac{\epsilon }{\theta }\sqrt{\min {c_1, c_2}}\quad & \textrm{if}\ (\alpha _2, \alpha _3, \alpha _4)= (0,1,0),\ k= 0 \end{array} \right. \end{aligned}$$

because of (12).

*Case* $\alpha _1=\alpha _3=0$. We obtain, from (40),

$$\begin{aligned} |\textrm{d}^1_{\alpha , k}(\lambda , \omega )|= &  \Big | (\alpha _2+\alpha _4) a_2-a_1 +\textrm{i}\Big ( -\Omega _1+(\alpha _2-\alpha _4)\Omega _2 +k\omega \Big )\Big |\nonumber \\ &  \textrm{if}\quad \alpha _1=\alpha _3=0 \end{aligned}$$

If $k\ne 0$, from the imaginary part, we have

42 $$\begin{aligned} |\textrm{d}^1_{\alpha , k}(\lambda , \omega )|\ge \omega -(2K+1)|\Omega |\ge \frac{\omega }{2} \end{aligned}$$

Otherwise, if $k=0$, from condition (14), with $\alpha =\alpha _2+\alpha _4$ and $q=\alpha _2-\alpha _4$, we have

43 $$\begin{aligned} |\textrm{d}^1_{\alpha , k}(\lambda , \omega )|\ge &  \frac{\upupsilon }{(2K+1)^{\tau }}. \end{aligned}$$

Collecting (39),  (41), (42) and (43), we obtain (38). With a cyclic permutation of the $\alpha$–indices, one obtains the proof for the cases $h=2$, 3, 4. $\square$

##### Proof of Theorem 2.1

We let $$\zeta _0:=(\gamma , p_\gamma , \psi , p_\psi )$$, $$\varphi _0:=\ell $$, $$x_0:=(\zeta _0, \varphi _0)$$.

44 $$\begin{aligned} N_0=\left( \begin{array}{cc}0\\ \omega \end{array}\right) ,\quad P_0:=\left( \begin{array}{cc}L\zeta _0\\ 0 \end{array}\right) +\breve{P}+{\widetilde{P}}+{\widehat{P}}\end{aligned}$$

where the matrix *L* as well as the components of $\breve{P}$, ${\widetilde{P}}$ and ${\widehat{P}}$ are defined via the right hand side of (7). Then the vector–field at right hand side of (7) is

45 $$\begin{aligned} X_0(x_0)=N_0+P_0(x_0) \end{aligned}$$

We next proceed in four steps. Along the proof, $\textrm{g}_1$, $\ldots$, $\textrm{g}_{12}$ will denote suitably large numbers, independent of $c_1$, $c_2$, $\epsilon$, $\delta$, $\upupsilon$, $v_0$, *r*, *a*, *C*, $C'$.

*Step 1: application of Theorem *3.1*to* $X_0$ We fix $u=u_0=(\varepsilon _0, s_0)$ so that $P_0$ is real–analytic in the domain

$$\begin{aligned} \zeta _0\in B^4_{\varepsilon _0},\ \varphi _0\in {\mathbb {T}}_{s_0} \end{aligned}$$

and choose the weights $w_0=\frac{u_0}{4}$. We aim to apply Theorem 3.1 to $N=N_0$, $P=P_0$ as in (45). From equations (6) and (3), we can bound

46 $$\begin{aligned} |\!|\!|P_0|\!|\!|_{u_0}^{w_0}\le {{\, \mathrm g}_1}\max \left\{ c_1,\ c_2,\ \varepsilon _0,\ \epsilon \varepsilon _0,\ \delta \varepsilon _0,\ |v_0|\upupsilon ,\ \frac{r^2}{a^3\varepsilon _0\min \{C,\ C'\}} \right\} =:{{\, \mathrm g}_1}{\bar{\mu }}_0. \end{aligned}$$

Incidentally, remark that ${\bar{\mu }}_0$ is defined similarly as $\mu _0$ in (15), apart for the fact that the last term in the bracket is missing, whence ${\bar{\mu }}_0\le \mu _0$. A similar observation will hold for the parameter ${\bar{\mu }}_1\le \mu _1$, which will be introduced in Step 3 below. Condition (28) is satisfied, due to (16), provided that $c\ge C_*{\bar{\sigma }}\mathrm{g_1}$. We prove that, with the choice

47 $$\begin{aligned} {\gamma _0=\frac{\omega }{2}} \end{aligned}$$

we obtain

48 $$\begin{aligned} {\Lambda _{0h}}:=\Lambda _{h}^{\frac{\omega }{2}}={\mathbb {N}}^4\times \Big \{0\Big \}\quad \forall \ h=1,\ldots , 5. \end{aligned}$$

Indeed, as all the $\lambda _i$’s are zero, we have

$$\begin{aligned} \textrm{d}^h_{\alpha , k}(0, \omega )=-\textrm{i} k\omega \qquad \forall \ \alpha \in {\mathbb {N}}^4,\quad \forall \ h=1,\ldots , 5. \end{aligned}$$

Hence, as the Keplerian frequency $\omega$ does not vanish,

$$\begin{aligned} \Lambda _{h}^{{\frac{\omega }{2}}}={\mathbb {N}}^4\times \Big \{k\in {\mathbb {Z}}:\ |k|\omega \le {\frac{\omega }{2}}\Big \}{={\mathbb {N}}^4\times \Big \{0\Big \}}\quad \forall \ h=1,\ldots , 5. \end{aligned}$$

as claimed. We denote as $\Lambda _{0}=(\Lambda _{01},\ldots ,\Lambda _{05})$ the 5–ple of lattices (48). If $\tau _0$ corresponds to $\tau$ in the thesis of Theorem 3.1, we choose

49 $$\begin{aligned} K_0\ge {\tau _0}^{-1}\log \left( \frac{{\bar{\mu }}_0}{\omega }\right) ^{-1}. \end{aligned}$$

By the thesis of Theorem 3.1, we find a change of coordinates

50 $$\begin{aligned} \phi _1:\ x_1=(\zeta _1, \varphi _1)\in V_{u_1}\rightarrow x_0=(\zeta _0, \varphi _0)=\phi _1(\zeta _1, \varphi _1)\in V_{u_0} \end{aligned}$$

where $u_1=\frac{u_0}{2}$, which transforms the vector–field $X_0$ in (45) to

51 $$\begin{aligned} X_1(x_1)=N_0+ {\overline{P}}_0(\zeta _1)+\widetilde{P}_1(x_1)\in {\mathcal {O}}_{u_1} \end{aligned}$$

${\overline{P}}_0$ is the $\varphi _0$–average of $P_0$ (because in this case $G_+=\Pi _{{\Lambda }_0} T_{K_0} P_{0}={\overline{P}}_0$) and ${\widetilde{P}}_1(x_1)$, corresponding to $P_+$, verifies

$$\begin{aligned} |\!|\!|{\widetilde{P}}_1|\!|\!|_{u_0/2}^{u_0/4}\le {{\, \mathrm g}_2} \frac{{\bar{\mu }}_0^2}{\omega } \end{aligned}$$

having used (49). By (31), (46) and (47), the transformation $\phi _1$ in (50) verifies

52 $$\begin{aligned} |\phi _1-\textrm{id}|_{u_0/2}^{u_0/4}\le \frac{{\bar{\mu }}_0}{\omega }. \end{aligned}$$

*Step 2: diagonalization of the linear part* Taking the decomposition (44) of $P_0$ into account, the vector–field $X_1(x_1)$ in (51) can be written as

53 $$\begin{aligned} X_1(x_1)=N_1(\zeta _1)+P_1(x_1) \end{aligned}$$

where

$$\begin{aligned}N_1(\zeta _1)=\left( \begin{array}{cc}L\zeta _1\\ \omega \end{array}\right) ,\quad P_1(x_1):=\breve{P}(x_1)+\widehat{{\overline{P}}}(x_1)+{\widetilde{P}}_1(x_1) \end{aligned}$$

with $\widehat{{\overline{P}}}(\zeta _1)$ being the $\varphi _0$–average of ${\widehat{P}}$ computed in $\zeta _1$. Here, we have used that ${\widetilde{P}}$ has vanishing $\varphi _0$–average and $\breve{P}$, is $\varphi _0$–independent. In Sect. 5 it is shown that the eigenvalues of *L* are distinct and have negative real part. If *b* is the $4\times 4$ matrix such that $b^{-1}Lb$ we define the change of coordinates

$$\begin{aligned} \phi _2:\quad x_2=(\zeta _2, \varphi _2)\in V_{u_2}\rightarrow x_1=(\zeta _1, \varphi _1)=\phi _2(x_2):=(b\zeta _2, \varphi _2)\in V_{u_1}. \end{aligned}$$

with

$$\begin{aligned} u_2=(\varepsilon _2, s_2),\quad \varepsilon _2:=\frac{\varepsilon _1}{\Vert b\Vert },\qquad s_2:=s_1 \end{aligned}$$

with $\Vert b\Vert$ denoting the operator norm of *b*. The change $\phi _2$ carries the vector–field $X_1(x_1)$ in (53) to

54 $$\begin{aligned} X_2(x_2):=\phi _2^{-1}X_1(\phi _2(x_2))=N_2(\zeta _2)+P_2(x_2) \end{aligned}$$

where

55 $$\begin{aligned} N_2(\zeta _2)=\phi _2^{-1}N_1(b\zeta _2)=\left( \begin{array}{ccc} \lambda _1 \zeta _{2, 1}\\ \lambda _2 \zeta _{2, 2}\\ \lambda _3 \zeta _{2, 3}\\ \lambda _4\zeta _{2, 4}\\ \omega \end{array}\right) ,\qquad P_2(x_2):=\phi _2^{-1}P_1(\phi _2(x_2)).\end{aligned}$$

with $\lambda _j$ the eigenvalues of *L*.

*Step 3: application of Theorem* 3.2*to* $X_2$ Choosing $w_2:=\frac{u_2}{8}$, from (8), (44) and (55), we have

56 $$\begin{aligned} |\!|\!|P_2|\!|\!|_{u_2}^{u_2/8}\le {{\, \mathrm g}_3}\max \left\{ \frac{{\bar{\mu }}_0^2}{\omega },\ {\bar{\mu }}_0\frac{r}{a},\ c_1\varepsilon _0^3,\ c_2\varepsilon _0^2\right\} =:{{\, \mathrm g}_3}{\bar{\mu }}_1\end{aligned}$$

where, as anticipated in Step 1 above, ${\bar{\mu }}_1\le \mu _1$, with $\mu _1$ given in (15). Let

57 $$\begin{aligned} &  {K_1:=min\left\{ \omega \frac{\epsilon }{\theta }\left( max\{c_1, c_2\}\right) ^{-1},\ {\left( \upupsilon \frac{\theta }{\epsilon }\left( min\{c_1, c_2\}\right) ^{-1}\right) ^{\frac{1}{\tau }}},\ \left( \frac{\upupsilon }{{\bar{\mu }}_{{1}}}\right) ^{\frac{1}{\tau +1}}\right\} ,}\nonumber \\ &  \gamma _1:=\frac{\upupsilon }{(3K_{{1}})^\tau },\ \end{aligned}$$

It is readily checked that that inequalities (32), (33) and (35) are satisfied if $K=K_1$, $\gamma =\gamma _1$, provided that *c* is suitably large, as a consequence of the assumptions (16). By the validity of (32) and (33), Theorem 3.2 applies. By the validity of (35), one has

$$\begin{aligned} \Lambda _{1h}:=\Lambda ^{\gamma _1, 2K_1}_{h}=\{p_h\}\qquad h=1,2,3,4,5 \end{aligned}$$

with $p_h$ as in (21). By the thesis of Theorem 3.2, one finds a change of coordinates

58 $$\begin{aligned} \phi _3:\ x_3=(\zeta _3, \varphi _3)\in V_{u_3}\rightarrow x_2=(\zeta _2, \varphi _2)=\phi _3(\zeta _3, \varphi _3)\in V_{u_2} \end{aligned}$$

with $u_3=\frac{u_2}{2}$, which carries the vector–field $X_2(x_2)$ in (54) to

59 $$\begin{aligned} X_3(x_3)=N_3(\zeta _3)+P_3(x_3) \end{aligned}$$

where

60 $$\begin{aligned} N_3(\zeta _3)=N_2(\zeta _3)+G_3(\zeta _3) \end{aligned}$$

with $N_2$ as in (55), and $G_3$ satisfies $G_3=\Pi _{\Lambda _{1}}T_{K_{1}} G_3$. As $\Lambda _{1h}=\{p_h\}$, it follows that $G_3$ has the form

61 $$\begin{aligned} G_3(\zeta _3)=\left( \begin{array}{ccc} {\tilde{\lambda }}_1 \zeta _{3, 1}\\ {\tilde{\lambda }}_2 \zeta _{3, 2}\\ {\tilde{\lambda }}_3 \zeta _{3, 3}\\ {\tilde{\lambda }}_4\zeta _{3, 4}\\ {{\tilde{\omega }}} \end{array}\right) \end{aligned}$$

Moreover, the following bounds hold:

62 $$\begin{aligned} &  |\!|\!|G_3|\!|\!|_{u_3}^{u_3/4}\le |\!|\!|\Pi _{{\Lambda _{1}}} T_{K_{1}}P_2|\!|\!|_{u_3}^{u_3/4}+|\!|\!|G_3-\Pi _{{\Lambda _{1}}} T_{K_{1}}P_2|\!|\!|_{u_3}^{u_3/4} \le {{\, \mathrm g}_2}{\bar{\mu }}_1+ {{\, \mathrm g}_4}\frac{{\bar{\mu }}^2_1}{\gamma _1}\le {{\, \mathrm g}_5}{\bar{\mu }}_1 \nonumber \\ &  |\!|\!|P_3|\!|\!|_{u_3}^{u_3/4}\le |\!|\!|P_2|\!|\!|_{u_2}^{u_2/8} e^{-K_1{\bar{\sigma }}/4}\le {{\, \mathrm g}_6}{\bar{\mu }}_1 {e^{-K_1{\bar{\sigma }}/4}}. \end{aligned}$$

By (34), (56) and (36), the transformation $\phi _3$ in (58) verifies

63 $$\begin{aligned} |\phi _3-\textrm{id}|_{u_2/2}^{u_2/8}\le {{\, \mathrm g}_7}\frac{{\bar{\mu }}_1}{\gamma _1}{= \textrm{g}_8 K_1^\tau \frac{{\bar{\mu }}_1}{\upupsilon }\le \frac{\textrm{g}_9}{K_1}}. \end{aligned}$$

*Step 4: conclusion* By (62) and Lemma 4.1 below, the numbers ${\tilde{\lambda }}_j$ in (61) verify

64 $$\begin{aligned} |{\tilde{\lambda }}_j|= &  \left| \left. \partial _{\zeta _{3, j}}G_3(\zeta _3)\right| _{\zeta _{3, j}=0}\right| {\le \textrm{g}_{10}{\bar{\mu }}_1}\end{aligned}$$

Using (59), (60) and (61), we have that the coordinates $\zeta _{3, i}$ satisfy the ODEs

65 $$\begin{aligned} {\dot{\zeta }}_{3, j}={\hat{\lambda }}_j\zeta _{3, j}+P_{3, j}(\zeta _3, \omega t) \end{aligned}$$

where ${\hat{\lambda }_{j}}:=\lambda _j+{\tilde{\lambda }}_j$. Moreover, ${\hat{\lambda }_{j}}$ have negative real part, as it follows from (64) and the inequality, implied by (16),

$$\begin{aligned} {\bar{\mu }}_1\le \frac{\upupsilon }{3}\le \min _j|{\, \mathrm Re\, }\lambda _j|. \end{aligned}$$

Rewriting (65) in the form

$$\begin{aligned} \zeta _{3, j}(t)= \zeta _j(0)e^{{\hat{\lambda _jt}}}+\int _0^tP_{3, j}(\zeta _3(\tau ), \omega \tau )e^{{\hat{\lambda _j}}(t-\tau )}d\tau \end{aligned}$$

we find (since ${\, \mathrm Re\, }{\hat{\lambda _j}}<0$)

66 $$\begin{aligned} |\zeta _{3, j}(t)-\zeta _{3, j}(0)e^{{\hat{\lambda _j}} t}|= &  \left| \int _0^tP_{3, j}(\zeta _3(\tau ), \omega \tau )e^{{\hat{\lambda _j}}(t-\tau )}d\tau \right| \nonumber \\\le &  \int _0^{|t|}|P_{3, j}(\zeta _3(\tau ), \omega \tau )|d\tau \le {\textrm{g}_{11}} |t |{\bar{\mu }}_1 {e^{-K_1{\bar{\sigma }}/4}} \nonumber \\\le &  \varepsilon \qquad \forall \ |t|\le {\frac{\varepsilon }{\textrm{g}_{11}{\bar{\mu }}_1}}\, {e^{K_1{\bar{\sigma }}/4}}. \end{aligned}$$

On the other hand, taking track of the transformations, $x_0$ and $x_3$ are related via

67 $$\begin{aligned} {|x_0-\phi _2(x_3)|=|\phi _1\circ \phi _2\circ \phi _3(x_3)-\phi _2(x_3)|\le \textrm{g}_{12}\max \left\{ \frac{{\bar{\mu }}_0}{\omega },\ \frac{1}{K_1}\right\} \le \textrm{g}_{12}\varepsilon } \end{aligned}$$

having used (52) and (63) and the definitions of $\varepsilon$ and $K_1$ in (15) and (57). Taking the projection on $\zeta _3$, we find

$$\begin{aligned} |\zeta _0(t)-b \zeta _3(t)| \le \textrm{g}_{12}\varepsilon \qquad \forall \ t\in \mathbb {R}. \end{aligned}$$

Using finally (66), we arrive at (17), with $T={\frac{\varepsilon }{\textrm{g}_{11}{\bar{\mu }}_1}} \, {e^{K_1{\bar{\sigma }}/4}}$. On the other hand, the definition of $K_1$ in (57) and the last inequality in (67) imply

$$\begin{aligned} \frac{1}{{\bar{\mu }}_1}\ge \frac{K_1^{\tau +1}}{\upupsilon },\qquad K_1\ge \frac{1}{\varepsilon } \end{aligned}$$

whence the lower bound for *T* in (18) follows. The theorem is proved. $\square$

## Proof of Theorems 3.1 and 3.2

### Proof of Theorem 3.1

#### Definition 4.1

- We call *time–one flow of* *Y* a one–parameter of diffeomorphism $\Phi _1^Y$, where $x(\tau ):=\Phi ^Y_\tau (y)$ solves
  $$\begin{aligned}\left\{ \begin{array}{lll} \partial _{\tau } x=Y(x)\\ \\ x(0)=y \end{array} \right. \end{aligned}$$
- For a given $C^{\infty }$ vector–field *Y*, we denote as
  $$\begin{aligned}{\mathcal {L}}_Y:=[Y, \cdot ]\end{aligned}$$
  the *Lie operator*, where
  $$\begin{aligned}(Y, X):=J_X Y-J_Y X,\quad (J_X)_{ij}:=\partial _{x_j} X_i\end{aligned}$$
  denotes the *Lie brackets* of two vector–fields. The map
  68 $$\begin{aligned} e^{{\mathcal {L}}_Y}:=\sum _{{{k}}=0}^{+\infty }\frac{{\mathcal {L}}_Y^{{k}}}{{{k}}!}\end{aligned}$$
  is called *Lie series* generated by *Y*.

#### Proposition 4.1

Assume that $e^{{\mathcal {L}}_Y}$ is well defined. Then the time–one map of *Y*, $\Phi ^Y_1$, carries the ODE (25) to $\dot{y}=Z(y)$, where $Z=e^{{\mathcal {L}}_Y} X$.

Proposition 4.1 is a well–known result in differential geometry. A self–contained proof can be however found in Appendix A.

Our aim is now to provide conditions so that the series (68) is well defined.

#### Lemma 4.1

(Cauchy Inequalities) Let $Z\in {\mathcal {O}}_{u}$, $u=(\varepsilon , s)$,$0<\rho <\varepsilon$, $0<\sigma <s$.

$$\begin{aligned}\mathrm{(i)}\ \Vert \partial ^p_{\varphi _i}Z_h\Vert _{u-\sigma }\le \left( \frac{p}{e \sigma }\right) ^p \Vert Z\Vert _{u},\qquad \mathrm{(ii)}\ \Vert \partial ^p_{\zeta _i}Z_h\Vert _{\varepsilon -\rho , s}\le \frac{p!}{\rho ^p} \Vert Z_h\Vert _{u}\end{aligned}$$

#### Proof

(i) From the formula

$$\begin{aligned}\partial ^p_{\varphi _i}Z_h=\sum _{(\alpha , k)}z^h_{\alpha , k}\zeta ^\alpha (\textrm{i}k_i)^p e^{\textrm{i}k\cdot \varphi }\end{aligned}$$

we get

$$\begin{aligned} \Vert \partial ^p_{\varphi _i}Z_h\Vert _{u-\sigma }= &  \sum _{(\alpha , k)}|z^h_{\alpha , k}|\varepsilon ^{|\alpha |_1} |k_i|^p e^{|k|_1(s-\sigma )}\nonumber \\\le &  \sum _{(\alpha , k)}|z^h_{\alpha , k}|\varepsilon ^{|\alpha |_1} |k|_1^p e^{-|k|_1\sigma )}e^{|k|_1s}\nonumber \\\le &  \frac{1}{\sigma ^p}\sup _{x\ge 0}x^p e^{-x}\sum _{(\alpha , k)}|z^h_{\alpha , k}|\varepsilon ^{|\alpha |_1} e^{|k|_1s}\nonumber \\= &  \left( \frac{p}{e \sigma }\right) ^p \Vert Z_h\Vert _{u},. \end{aligned}$$

(ii) From the formula

$$\begin{aligned}\partial ^p_{\zeta _i}Z_h=\sum _{(\alpha , k):\ \alpha _i\ge p}z^h_{\alpha , k}\alpha _i(\alpha _i-1)\cdots (\alpha _i-p+1)\zeta _i^{\alpha _i-p}\prod _{j\ne i}\zeta _j^{\alpha _j} e^{\textrm{i}k\cdot \varphi }\end{aligned}$$

we get

$$\begin{aligned} \Vert \partial ^p_{\zeta _i}Z_h\Vert _{\varepsilon -\rho , s}= &  \sum _{(\alpha , k):\ \alpha _i\ge p}|z^h_{\alpha , k}|\alpha _i(\alpha _i-1)\cdots (\alpha _i-p+1)(\varepsilon -\rho )^{\alpha _i-p} (\varepsilon -\rho )^{|{\hat{\alpha _i}}|_1} e^{|k|_1s}\nonumber \\= &  \frac{p!}{\rho ^p}\sum _{(\alpha , k):\ \alpha _i\ge p}|z^h_{\alpha , k}|\frac{\alpha _i(\alpha _i-1)\cdots (\alpha _i-p+1)}{p!}(\varepsilon -\rho )^{\alpha _i-p} \rho ^p \\  &  \quad (\varepsilon -\rho )^{|{\hat{\alpha _i}}|_1} e^{|k|_1s} \end{aligned}$$

with ${\hat{\alpha _i}}$ being $\alpha$ deprived if $\alpha _i$. Using now

$$\begin{aligned}\frac{\alpha _i(\alpha _i-1)\cdots (\alpha _i-p+1)}{p!}(\varepsilon -\rho )^{\alpha _i-p} \rho ^p &  \le \sum _{p=0}^{\alpha _i} \frac{\alpha _i(\alpha _i-1)\cdots (\alpha _i-p+1)}{p!}\\  &  \quad (\varepsilon -\rho )^{\alpha _i-p} \rho ^p=\varepsilon ^{\alpha _i}\end{aligned}$$

we get the thesis. $\square$

#### Lemma 4.2

Let $$w<u\le u_0$$; $$Y\in {\mathcal {O}}_{u_0}$$, $$W\in {\mathcal {O}}_{u}$$. Then

$$\begin{aligned}|\!|\!|{\mathcal {L}}_Y[W]|\!|\!|^{u_0-u+w}_{u-w}\le |\!|\!|Y|\!|\!|^{w}_{u-w}|\!|\!|W|\!|\!|^{u_0-u+w}_{u}+|\!|\!|W|\!|\!|^{u_0-u+w}_{u-w}|\!|\!|Y|\!|\!|^{u_0-u+w}_{u_0}. \end{aligned}$$

#### Proof

One has

$$\begin{aligned} |\!|\!|{\mathcal {L}}_Y[W]|\!|\!|^{u_0-u+w}_{u-w}&=|\!|\!|J_W Y-J_Y W|\!|\!|^{u_0-u+w}_{u-w}\nonumber \\&\le |\!|\!|J_W Y|\!|\!|^{u_0-u+w}_{u-w}+|\!|\!|J_Y W|\!|\!|^{u_0-u+w}_{u-w} \end{aligned}$$

Now, $(J_W Y)_i=\sum _{j}\partial _{x_j} W_i Y_j$, so, using Cauchy inequalities,

$$\begin{aligned} \Vert (J_W Y)_i\Vert _{u-w}&\le \sum _{j}\Vert \partial _{x_j} W_i\Vert _{u-w}\Vert Y_j\Vert _{u-w}\nonumber \\&\le \sum _{j}w_j^{-1}\Vert W_i\Vert _{u}\Vert Y_j\Vert _{u-w}\nonumber \\&=|\!|\!|Y|\!|\!|^w_{u-w}\Vert W_i\Vert _{u} \end{aligned}$$

Similarly,

$$\begin{aligned}\Vert (J_Y W)_i\Vert _{u-w}\le |\!|\!|W|\!|\!|_{u-w}^{u_0-u+w}\Vert Y_i\Vert _{u_0}. \end{aligned}$$

Taking the $u_0-u+w$–weighted norms, the thesis follows. $\square$

#### Lemma 4.3

Let $$0<w<u$$, $$Y\in {\mathcal {O}}_{u+w}$$, $$W\in {\mathcal {O}}_{u}$$. Then

69 $$\begin{aligned} |\!|\!|{\mathcal {L}}^{{k}}_Y[W]|\!|\!|^{w}_{u-w}\le {{k}}!q^k |\!|\!|W|\!|\!|^w _{u},\qquad q:=e |\!|\!|Y|\!|\!|^w_{u+w} \end{aligned}$$

#### Proof

We apply Lemma 4.2 with *W* replaced by ${\mathcal {L}}^{i-1}_Y[W]$, *u* replaced by $u-(i-1)w/{{k}}$, *w* replaced by *w*/*k* and, finally, $u_0=u+w$. With $|\!|\!|\cdot |\!|\!|_i^{w}=|\!|\!|\cdot |\!|\!|^{w}_{u-i\frac{w}{{{k}}}}$, $0\le i\le {{k}}$, so that $|\!|\!|\cdot |\!|\!|^w_0=|\!|\!|\cdot |\!|\!|^w_{u}$ and $|\!|\!|\cdot |\!|\!|^w_{{k}}=|\!|\!|\cdot |\!|\!|^w_{u-w}$,

$$\begin{aligned} |\!|\!|{\mathcal {L}}^i_Y[W]|\!|\!|^{w+w/{{k}}}_i= &  \left|\!|\!|\left[ Y, {\mathcal {L}}^{i-1}_Y[W]\right] \right|\!|\!|^{w+w/{{k}}}_i\nonumber \\\le &  |\!|\!|Y|\!|\!|^{w/{{k}}}_{i}|\!|\!|{\mathcal {L}}^{i-1}_Y[W]|\!|\!|^{w+w/{{k}}}_{i-1}+ |\!|\!|Y|\!|\!|^{w+w/{{k}}}_{u+w}|\!|\!|{\mathcal {L}}^{i-1}_Y[W]|\!|\!|^{w+w/{{k}}}_{i}. \end{aligned}$$

Hence, de–homogenizating,

$$\begin{aligned} \frac{{{k}}}{{{k}}+1}|\!|\!|{\mathcal {L}}^i_Y[W]|\!|\!|^{w}_i&\le {{k}} \frac{{{k}}}{{{k}}+1}|\!|\!|Y|\!|\!|^{w}_{i}|\!|\!|{\mathcal {L}}^{i-1}_Y[W]|\!|\!|^{w}_{i-1}+ \frac{{{k}}^2}{({{k}}+1)^2}|\!|\!|Y|\!|\!|^{w}_{u+w}|\!|\!|{\mathcal {L}}^{i-1}_Y[W]|\!|\!|^{w}_{i}\nonumber \\&\le \frac{{{k}}^2}{{{k}}+1}\left( 1+\frac{1}{{{k}}+1}\right) |\!|\!|Y|\!|\!|^{w}_{u+w}|\!|\!|{\mathcal {L}}^{i-1}_Y[W]|\!|\!|^{w}_{i-1} \end{aligned}$$

Eliminating the common factor $\frac{{{k}}}{{{k}}+1}$

$$\begin{aligned} |\!|\!|{\mathcal {L}}^i_Y[W]|\!|\!|^{w}_i&\le k\left( 1+\frac{1}{{{k}}+1}\right) |\!|\!|Y|\!|\!|^{w}_{u+w}|\!|\!|{\mathcal {L}}^{i-1}_Y[W]|\!|\!|^{w}_{i-1} \end{aligned}$$

and iterating *k* times from $i={{k}}$ to $i=1$, by Stirling, we get

$$\begin{aligned} |\!|\!|{\mathcal {L}}^{{k}}_Y[W]|\!|\!|^w _{u-w}&\le {{k}}^{{k}}\left( 1+\frac{1}{{k}}\right) ^{{k}}\left( |\!|\!|Y|\!|\!|^w_{u+w}\right) ^{{k}}|\!|\!|W|\!|\!|^w _{u}\le e^{{k}} {{k}}!\left( |\!|\!|Y|\!|\!|^w_{u+w}\right) ^{{k}}|\!|\!|W|\!|\!|^w _{u} \end{aligned}$$

as claimed. $$\square $$

Lemma 4.3 has the following immediate corollary. We denote as

70 $$\begin{aligned} e^{{\mathcal {L}}_Y}_m=\sum _{{{k}}\ge m}\frac{{\mathcal {L}}^{{k}}_Y}{{{k}}!} \end{aligned}$$

the *m*–tails of the Lie operator (68).

#### Proposition 4.2

Let $0<w<u$, $Y\in {\mathcal {O}}_{u+w}$, *q* as in (69) verify $0\le q<1$. Then the Lie series $e^{{\mathcal {L}}_Y}$ defines an operator

$$\begin{aligned}e^{{\mathcal {L}}_Y}:\quad {\mathcal {O}}_{u}\rightarrow {\mathcal {O}}_{u-w}\end{aligned}$$

and its *m*–tails (70) verify

$$\begin{aligned} \left|\!|\!|e^{{\mathcal {L}}_Y}_m W\right|\!|\!|^w_{u-w}\le \frac{q^m}{1-q}|\!|\!|W|\!|\!|_{u}^w\qquad \forall \ W\in {\mathcal {O}}_{u}. \end{aligned}$$

#### Definition 4.2

*(Homological equation)* We call *homological equation associated to* *N* an equation of the form

71 $$\begin{aligned} (Y, N)=Z. \end{aligned}$$

$$\begin{aligned} {{\mathcal {N}}}^{\, \gamma }_u:=\{Z'\in {{\mathcal {O}}}_u:\ \Pi _{\Lambda ^\gamma }Z'=0\}.\end{aligned}$$

#### Proposition 4.3

(i) If (71) holds with *Y*, $Z\in {{\mathcal {O}}}_u$, then $Z\in {{\mathcal {N}}}^0_u$.
(ii) Let $\gamma >0$ and $Z\in {{\mathcal {N}}}^{\, \gamma }_u$. Then there exists a unique $Y\in {{\mathcal {N}}}^{\, \gamma }_u$ verifying (71).
(iii) The unique vector–field *Y* in (ii) verifies
  $$\begin{aligned}\Vert Y_h\Vert _{u}\le \frac{\Vert Z_h\Vert _{u}}{\gamma }\qquad \forall \ h=1,\ \ldots , m+n.\end{aligned}$$

#### Proof

(i) The Jacobian of *N* is given by

$$\begin{aligned}J_N=\left( \begin{array}{cccccc} D_{m\times m}& 0_{m\times n}& \\ 0_{n\times m}& 0_{n\times n} \end{array} \right) \end{aligned}$$

where *D* is the diagonal matrix having the $\lambda _i$’s along its principal diagonal. Then we have

$$\begin{aligned}({{\mathcal {L}}}_YN)_h=[Y, N]_h=\Big (J_N Y-J_Y N(x)\Big )_h= \begin{array}{lllllll}a_h Y_h-\sum _{j=1}^{m}\lambda _jz_j\partial _{z_j}Y_h-\sum _{i=1}^{n}\omega _i\partial _{\varphi _i}Y_h \end{array} \end{aligned}$$

with $a_h=\lambda _h$ if $1\le h\le m$; $a_h=0$ if $m+1\le h\le m+n$. From these formulae one easily finds that the Taylor–Fourier expansion of the function $Z:={{\mathcal {L}}}_YN$ is related to corresponding one of *Y* via the relation among coefficients

72 $$\begin{aligned} z_{\alpha k}^{h}=\textrm{d}_{\alpha k}^{h}(\lambda , \omega )\, y_{\alpha k}^h\end{aligned}$$

with $\textrm{d}_{\alpha k}^{h}(\lambda , \omega )$ as in (22). But by definition of $\Lambda ^0_h$, $\textrm{d}_{\alpha k}^{h}(\lambda , \omega )=0$ for all $(\alpha , k)\in \Lambda ^0_h$. By (72), we then have also $z_{\alpha k}^{h}$ for all $(\alpha , k)\in \Lambda ^0_h$, which amounts to say $\Pi _{\Lambda ^0_h}Z_h=0$ for all $h=1$, $\ldots$, $m+n$, namely, $\Pi _{\Lambda ^0}Z=0$. As $Z\in {{\mathcal {O}}}_u$ by assumption, we then have $Z\in {{\mathcal {N}}}^0_u$.

(ii) Let $\gamma >0$ and $Z\in {{\mathcal {N}}}^\gamma _u$. Consider the vector–field *Y* defined via

73 $$\begin{aligned} Y_h=\sum _{(\alpha , k)\in {\mathbb {N}}^n\times {\mathbb {Z}}^n\setminus {\Lambda }^\gamma _h} \frac{z_{\alpha k}^h}{\textrm{d}^h_{\alpha k}}\zeta ^\alpha e^{\textrm{i}(k\cdot \varphi )}\end{aligned}$$

$Y_h$ is well defined because, due to the definition of $\Lambda ^\gamma _{h}$, we have

74 $$\begin{aligned} ||\textrm{d}^h_{\alpha k}(\lambda , \omega )|>\gamma \quad \forall \ (\alpha , k)\in {\mathbb {N}}^n\times {\mathbb {Z}}^n\setminus {\Lambda }^\gamma _h. \end{aligned}$$

Hence,

75 $$\begin{aligned} \Vert Y_h\Vert _{u}= &  \sum _{(\alpha , k)\in {\mathbb {N}}^n\times {\mathbb {Z}}^n\setminus {\Lambda }^\gamma _h } \frac{|z_{\alpha k}^h|}{|\textrm{d}^h_{\alpha k}|}\varepsilon ^{|\alpha |_1} e^{|k|_1 s}\le \gamma ^{-1}\sum _{(\alpha , k)\in {\mathbb {N}}^n\times {\mathbb {Z}}^n\setminus {\Lambda }^\gamma _h} {|z_{\alpha k}^h|}\varepsilon ^{|\alpha |_1} e^{|k|_1 s}\nonumber \\ = &  \gamma ^{-1}{\Vert Z_h\Vert _{u}}. \end{aligned}$$

which shows that $Y\in {{\mathcal {O}}}_u$. Moreover, by the discussion in (i), the Taylor–Fourier components of $Z'_h:=[Y, N]_h$ are obtained from the corresponding ones of $Y_h$ by a multiplication by $\textrm{d}^h_{\alpha k}$. Using (73) gives

$$\begin{aligned} [Y, N]_h=\sum _{(\alpha , k)\in {\mathbb {N}}^n\times {\mathbb {Z}}^n\setminus {\Lambda }^\gamma _h} {z_{\alpha k}^h}\zeta ^\alpha e^{\textrm{i}(k\cdot \varphi )} \end{aligned}$$

But the right hand side of this equality coincides with $Z_h$, as we have assumed $Z\in {{\mathcal {N}}}^\gamma$, whence $\Pi _{\Lambda _h}Z_h=0$ for all $h=1,\ \ldots , m+n$. Incidentally, we also proved (iii) in (75). $\square$

#### Definition 4.3

*(Ultraviolet* *K–tail)* Let $K\in { \mathbb {N}}$, $K>0$. We say that the vector–field $Z\in {{\mathcal {O}}}_u$ is a *ultraviolet K–tail* if

$$\begin{aligned}T_{K}Z=0\end{aligned}$$

with $T_KZ$ defined as in (24). The set of $Z\in {{\mathcal {O}}}_u$ for which this holds will be denoted as ${{\mathcal {T}}}^K_u$.

#### Lemma 4.4

(Estimate of the ultraviolet *K*–tail) Let $u=(\varepsilon , s)$, $w=(\rho , \sigma )<u$. Let $Z\in {\mathcal {T}}^{2K}_u$ be a ultraviolet 2*K*–tail. Then

76 $$\begin{aligned} \Vert Z_h\Vert _{u-w}\le e^{-K\tau }\Vert Z_h\Vert _{u},\qquad \tau :=\min \left\{ \sigma ,\ \log (1-\frac{\rho }{\varepsilon })^{-1}\right\} .\end{aligned}$$

#### Proof

By definition,

$$\begin{aligned}\Vert Z_h\Vert _{u-w}=\sum _{|(\alpha , k)|_1\ge 2K} |z_{\alpha k}^h|(\varepsilon -\rho )^{|\alpha |_1} e^{|k|_1 (s-\sigma )}\end{aligned}$$

Now, as $|(\alpha , k)|_1=|\alpha |_1+|k|_1$, either $|\alpha |_1\ge K$, or $|k|_1\ge K$. The terms of the summand with $|\alpha |_1\ge K$ are above by $(1-\frac{\rho }{\varepsilon })^K |z_{\alpha k}^h|\varepsilon ^{|\alpha |_1} e^{|k|_1 s}$; the ones with $|k|_1\ge K$ are bounded by $e^{-K\sigma }|z_{\alpha k}^h|\varepsilon ^{|\alpha |_1} e^{|k|_1 s}$. $\square$

#### Lemma 4.5

The norms (20) verify

$$\begin{aligned} |X|_u^w\le |\!|\!|X|\!|\!|_u^w\qquad \forall \ X\in {{\mathcal {O}}}_u,\ \forall \ 0<w<u. \end{aligned}$$

#### Proof

Obvious.

#### Theorem 4.1

Let $G\in {{\mathcal {O}}}_u$ verify $G=\Pi _{{\Lambda }^\gamma }T_{2K}G$. The thesis of Theorem 3.1 holds also if *X* in (26) is replaced with

$$\begin{aligned}X=N+G +P\in {\mathcal {O}}_{u}\end{aligned}$$

$$G_+$$ in (29) with

$$\begin{aligned}G_+=G+\Pi _{{\Lambda }^\gamma } T_{2K} P.\end{aligned}$$

and the inequality (30) with

$$\begin{aligned} |\!|\!|P_+|\!|\!|^{w}_{u-2w} \le \frac{1 }{1-e \gamma ^{-1} \left|\!|\!|P\right|\!|\!|^w_{u}}\left( e \gamma ^{-1}|\!|\!|P|\!|\!|^w_{u}|\!|\!|P|\!|\!|^w_{u-w}+\left|\!|\!|[Y,\ G]\right|\!|\!|^w_{u-w} +e^{-K \tau }\left|\!|\!|P\right|\!|\!|^w_{u}\right) \end{aligned}$$

#### Proof

We decompose

$$\begin{aligned}P=P^{<2K}+P^{\ge 2K}\end{aligned}$$

and, further,

$$\begin{aligned}P^{<2K}=\bar{P}+\tilde{P}\end{aligned}$$

with

$$\begin{aligned}P^{<2K}:=T_{2K}P,\qquad P^{\ge 2K}:=(I-T_{2K})P.\end{aligned}$$

and

$$\begin{aligned}\bar{P}:=\Pi _{{{\Lambda }^\gamma }}P^{<2K},\quad \tilde{P}= {(I-\Pi _{\Lambda ^\gamma })P^{<2K}}.\end{aligned}$$

We have

77 $$\begin{aligned} X_+= &  e^{{\mathcal {L}}_Y} X=e^{{\mathcal {L}}_Y}\left( N +G+P^{<2K}+P^{\ge 2K}\right) =N+G +P^{<2K}+[Y, N]+P_+\nonumber \\= &  N+G+\bar{P} +[Y, N]+\tilde{P}+P_+ \end{aligned}$$

with

78 $$\begin{aligned} P_+=e_2^{{\mathcal {L}}_Y} N +e_1^{{\mathcal {L}}_Y}P^{<2K}+e_1^{{\mathcal {L}}_Y}G+e_0^{{\mathcal {L}}_Y}P^{\ge 2K}\end{aligned}$$

By definition, $\Pi _{\Lambda ^\gamma }\tilde{P}=0$. Moreover, as $P\in {{\mathcal {O}}}_u$, also $\tilde{P}\in \mathcal{O}_u$. Then $\tilde{P}\in {{\mathcal {N}}}^\gamma _u$. By Proposition 4.3, (ii), there exists a unique $Y\in {\mathcal {N}}^\gamma _{u}$ verifying

79 $$\begin{aligned} (Y, N)=-\tilde{P}.\end{aligned}$$

Then (77) becomes

$$\begin{aligned}X_+=N+G_++P_+ \end{aligned}$$

with $G_+:=G +\bar{P}$. The time–one flow of *Y* is well defined as per Proposition 4.2, because.

80 $$\begin{aligned} q:=e |\!|\!|Y|\!|\!|^{w} _{u}&\le e \gamma ^{-1}|\!|\!|\tilde{P}|\!|\!|^w_u\le e \gamma ^{-1} |\!|\!|P|\!|\!|^w_u<1. \end{aligned}$$

By Proposition 4.2, the Lie series $e^{{\mathcal {L}}_Y}$ defines an operator

$$\begin{aligned}e^{{\mathcal {L}}_Y}:\quad W\in {\mathcal {O}}_{u-w}\rightarrow {\mathcal {O}}_{u-2w}\end{aligned}$$

and its tails $e^{{\mathcal {L}}_Y}_m$ verify

$$\begin{aligned} \left|\!|\!|e^{{\mathcal {L}}_Y}_m W\right|\!|\!|^{w}_{u-2w}&\le \frac{q^m}{1-q}|\!|\!|W|\!|\!|^{w}_{u-w}\nonumber \\&\le \frac{\left( e\gamma ^{-1}|\!|\!|P|\!|\!|^w_u\right) ^m}{1-e\gamma ^{-1}|\!|\!|P|\!|\!|^w_u}|\!|\!|W|\!|\!|^{w}_{u-w} \end{aligned}$$

for all $W\in {\mathcal {O}}_{u-w}$. In particular, $e^{{\mathcal {L}}_Y}$ is well defined on ${\mathcal {O}}_{u-w}\subset {\mathcal {O}}_{u}$, hence $P_+\in {\mathcal {O}}_{u-w}$. The bounds on $P_+$ in (78) are obtained as follows. Using the homological equation (79), one finds

$$\begin{aligned} e_2^{{\mathcal {L}}_Y}N+e_1^{{\mathcal {L}}_Y}P^{<2K}= &  \sum _{{{k}}=1}^{\infty }\frac{{\mathcal {L}}^{{{k}}+1}_Y N}{({{k}}+1)!}+ \frac{{\mathcal {L}}^{{{k}}}_Y P^{<2K}}{{{k}}!}\nonumber \\= &  \sum _{{{k}}=1}^{\infty }{\mathcal {L}}^{{{k}}}_Y\left( -\frac{\tilde{P}}{({{k}}+1)!}+ \frac{ P^{<2K}}{{{k}}!}\right) \nonumber \\= &  \sum _{{{k}}=1}^{\infty }{\mathcal {L}}^{{{k}}}_Y\left( \frac{k}{({{k}}+1)!}\tilde{P}+ \frac{\bar{P}}{{{k}}!}\right) \end{aligned}$$

which gives

$$\begin{aligned} |\!|\!|e_2^{{\mathcal {L}}_Y}N+e_1^{{\mathcal {L}}_Y}P^{<2K}|\!|\!|_{u-w}^w\le &  \sum _{{{k}}=1}^{\infty } q^k k!\left|\!|\!|\frac{k}{({{k}}+1)!}\tilde{P}+ \frac{\bar{P}}{{{k}}!}\right|\!|\!|_{u-w}^w\nonumber \\= &  \sum _{{{k}}=1}^{\infty } q^k k!\left( \frac{k}{({{k}}+1)!}|\!|\!|\tilde{P}|\!|\!|_{u-w}^w+ \frac{1}{{{k}}!}|\!|\!|{\bar{P}}|\!|\!|_{u-w}^w\right) \nonumber \\\le &  \sum _{{{k}}=1}^{\infty } q^k|\!|\!|P^{<2K}|\!|\!|_{u-w}^w=\frac{q}{1-q}|\!|\!|P^{<2K}|\!|\!|_{u-w}^w \end{aligned}$$

The other bounds

$$\begin{aligned} &  \left|\!|\!|e_1^{{\mathcal {L}}_Y}G\right|\!|\!|^{w}_{u-2w} \le \frac{1}{1-q}\left|\!|\!|{\mathcal {L}}_Y G\right|\!|\!|^w_{u-w}=\frac{1}{1-q}\left|\!|\!|[Y,\ G]\right|\!|\!|^w_{u-w}\nonumber \\ &  \left|\!|\!|e_0^{{\mathcal {L}}_Y}P^{\ge 2K}\right|\!|\!|^{w}_{u-2w} \le \frac{1}{1-q}\left|\!|\!|P^{\ge 2K}\right|\!|\!|^w_{u-w}\le \frac{1}{1-q}e^{-K \tau }\left|\!|\!|P\right|\!|\!|^w_{u} \end{aligned}$$

are similarly established. Finally, it follows from the identity

$$\begin{aligned} \phi _+(x_+)=\Phi _1^Y(x_+)=x_++Y(\Phi _{\tau _*}^Y(x_+))\qquad \tau _*\in (0, 1) \end{aligned}$$

and Lemma 4.5 that

$$\begin{aligned} |\phi _+-\textrm{id}|_{\bar{u}}^{\bar{w}}\le |Y|_{\bar{u}}^{\bar{w}}\le |\!|\!|Y|\!|\!|_{\bar{u}}^{\bar{w}}\le |\!|\!|Y|\!|\!|_{u}^{\bar{w}} \quad \forall \ \bar{u}\le u:\ \Phi _{\tau _*}^Y(x_+)\in U_{\bar{u}},\ \forall \bar{w} \end{aligned}$$

Taking $\bar{u}=u-2w$, $\bar{w}=w$ and using (80), we have

$$\begin{aligned} |\phi _+-\textrm{id}|_{u-2w}^w\le |Y|^w_u\le |\!|\!|Y|\!|\!|^w_u \le \gamma ^{-1} |\!|\!|P|\!|\!|^w_u \end{aligned}$$

which is (31). $$\square $$

### Proof of Theorem 3.2

Put

$$\begin{aligned}x=x_0:=(\zeta _0, \varphi _0),\qquad X_0(x_0):=N(\zeta _0)+P_0(x_0). \end{aligned}$$

We aim to apply Theorem 4.1 to $X_0$ hence, with $G_0=0$. This is possible because non–resonance condition is verified, and the inequalities (32) and (33) imply (28), provided that $C_*\log (12)\ge e$. We then find $Y_0\in {\mathcal {O}}_{u}$ such that $\phi _1:=\Phi _1^{Y_0}$ and $\Phi _0=e^{{\mathcal {L}}_{Y_0}}$ verify

81 $$\begin{aligned} \phi _{1}:\quad x_1\in V_{u-2w}\rightarrow x_{0}\in V_{u_{0}},\quad \Phi _0:\ {\mathcal {O}}_{u}\rightarrow {\mathcal {O}}_{u-2w}\end{aligned}$$

such that

82 $$\begin{aligned} X_1:=e^{{{\mathcal {L}}}_{Y_0}} X_0=N+\bar{P}_0+P_1\end{aligned}$$

where

83 $$\begin{aligned} \bar{P}_0\in {\mathcal {O}}_u\end{aligned}$$

and

84 $$\begin{aligned} |\!|\!|P_1|\!|\!|^{w}_{u-2w}\le &  \frac{1 }{1-e \gamma ^{-1} \left|\!|\!|P_0\right|\!|\!|^w_{u}}\left( e \gamma ^{-1}|\!|\!|P_0|\!|\!|^w_{u}|\!|\!|P_0|\!|\!|^w_{u-w}+e^{-K \tau }\left|\!|\!|P_0\right|\!|\!|^w_{u}\right) \nonumber \\\le &  2\left|\!|\!|P_0\right|\!|\!|^w_{u}\left( e \gamma ^{-1}|\!|\!|P_0|\!|\!|^w_{u}+e^{-K \tau }\right) \end{aligned}$$

If $\gamma ^{-1}|\!|\!|P_0|\!|\!|^w_{u}\le e^{-K \tau }$, there is no much to say. Indeed, using

$$\begin{aligned}\tau =\min \left\{ \sigma ,\ \log \left( 1-\frac{\rho }{\varepsilon }\right) ^{-1}\right\} \ge \min \left\{ \sigma ,\ \frac{\rho }{\varepsilon }\right\} = {\bar{\sigma }}\end{aligned}$$

and (32), we have

$$\begin{aligned} |\!|\!|P_1|\!|\!|^{w}_{u-2w} \le 4e^{-K \tau }\left|\!|\!|P_0\right|\!|\!|^w_{u}=e^{-K \tau +2\log 2}\left|\!|\!|P_0\right|\!|\!|^w_{u}\le e^{-K{\bar{\sigma }}+2\log 2}\left|\!|\!|P_0\right|\!|\!|^w_{u}\le e^{-K {\bar{\sigma }}/4}\left|\!|\!|P_0\right|\!|\!|^w_{u}\end{aligned}$$

and the proof ends here. If, instead, $\gamma ^{-1}|\!|\!|P_0|\!|\!|^w_{u}> e^{-K \tau }$, we need a recursion.

Fix

85 $$\begin{aligned} p\in { \mathbb {N}}\setminus \{0\},\qquad p\le \frac{K{\bar{\sigma }}}{\log (12)}\end{aligned}$$

By (32), such a *p* exist. The number *p* will be used as the amount of iterations. The higher bound in the second inequality in (85) will be needed in order to guarantee a suitably fast decay of the perturbing terms. Later on, we shall choose *p* as the greatest natural number satisfying such inequality, but this is not needed as of now. As of now, we observe that combining such inequality with condition (33), we have

86 $$\begin{aligned} ep C \gamma ^{-1} |\!|\!|P|\!|\!|^{w} _{u}<1\qquad \end{aligned}$$

with $C:=e^{-1}C_*\log (12)$. A suitable $C\ge 1$ (which corresponds to a suitable $C_*\ge e/\log (12)$) will be fixed along the way.

*Induction* We prove that, if

$$\begin{aligned}u_0:=u,\quad w_0:=w,\quad u_j=u-2w-2\frac{j-1}{p}w,\quad w_j=\frac{w}{p}\qquad j\in \{1,\ldots ,\ p+1\}\end{aligned}$$

for any $j\in \{1,\ldots ,\ p+1\}$, it is possible to find $Y_{j-1}\in {{\mathcal {O}}}_{u_{j-1}}$ such that $\phi _{j}=\Phi ^{Y_{j-1}}_1$ and $\Phi _{j-1}:=e^{{{\mathcal {L}}}_{Y_{j-1}}}$ verify

87 $$\begin{aligned} \phi _{j}:\quad x_j\in V_{u_j}\rightarrow x_{j-1}\in V_{u_{j-1}},\quad \Phi _{j-1}:\ {\mathcal {O}}_{u_{j-1}}\rightarrow {\mathcal {O}}_{u_{j}}\end{aligned}$$

and

88 $$\begin{aligned} X_j=\Phi _{j-1}X_{j-1}=N+\sum _{i=0}^{j-1} \bar{P}_i+P_j\end{aligned}$$

where

89 $$\begin{aligned} &  \bar{P}_{i}\in {\mathcal {O}}_{u_{i}}\quad \forall \ 0\le i\le j-1, \end{aligned}$$

90 $$\begin{aligned} &  |\!|\!|P_j|\!|\!|^{w}_{u_j}\le \frac{1}{2}|\!|\!|P_{j-1}|\!|\!|^w_{u_{j-1}}\end{aligned}$$

and, moreover,

91 $$\begin{aligned} e\gamma ^{-1}|\!|\!|P_j|\!|\!|^{w/p}_{u_j}<1.\end{aligned}$$

When $j=1$, (87), (88) and (89) are precisely as in (81), (82) and (83). We check that also (90), (91) are true with $j=1$. Indeed, (86) and (84) imply

92 $$\begin{aligned} |\!|\!|P_1|\!|\!|^{w}_{u-2w} \le 4 e \gamma ^{-1}\left( |\!|\!|P_0|\!|\!|^w_{u}\right) ^2\le \frac{1}{2}|\!|\!|P_0|\!|\!|^w_{u}\quad (C\ge 8) \end{aligned}$$

and, moreover,

93 $$\begin{aligned} e\gamma ^{-1}|\!|\!|P_1|\!|\!|^{w/p}_{u-2w}=e\gamma ^{-1}|\!|\!|P_1|\!|\!|^{w}_{u-2w}p \le 4\left( e \gamma ^{-1}|\!|\!|P_0|\!|\!|^w_{u} \right) ^2p<\frac{4}{C^2p}<1\end{aligned}$$

so the base step $j=1$ is complete. Let us now assume that (87), (88), (89), (90), (91) hold for some $j\in \{1,\ \ldots ,\ p\}$, and let us prove the same for $j+1$.

By (91) and the non–resonance condition, Theorem 4.1 can be applied with $X=X_j$, $G=\sum _{i=0}^{j-1}\bar{P}_i$, $P=P_j$, $u=u_j$, *w* replaced by *w*/*p* and one finds $\Phi _j$ verifying (87), (88), (89) with *j* replaced by $j+1$.

We prove that (90) holds with *j* replaced by $j+1$. This will end the induction, after remarking that  (91) with *j* replaced by $j+1$ is trivially implied by  (91) itself and (90) with *j* replaced by $j+1$. By the thesis of Theorem 4.1, we have

$$\begin{aligned} &  |\!|\!|P_{j+1}|\!|\!|^{w/p}_{u_{j+1}} \le \frac{1 }{1-e \gamma ^{-1} \left|\!|\!|P_j\right|\!|\!|^{w/p}_{u_j}} \nonumber \\ &  \left( e \gamma ^{-1}|\!|\!|P_j|\!|\!|^{w/p}_{u_j}|\!|\!|P_j|\!|\!|^{w/p}_{u_j-w/p}+ |\!|\!|[ Y_j,\ \sum _{i=0}^{j}\bar{P}_i ]|\!|\!|^{w/p}_{u_j-w/p} +e^{-K \tau (p)}|\!|\!|P_j|\!|\!|^{w/p}_{u_j}\right) \nonumber \\\le &  2\left|\!|\!|P_j\right|\!|\!|^{w/p}_{u_j}\left( e \gamma ^{-1}|\!|\!|P_j|\!|\!|^{w/p}_{u_j}+e^{-K \tau (p)}\right) +2|\!|\!|[ Y_j,\ \sum _{i=0}^{j}\bar{P}_i ]|\!|\!|^{w/p}_{u_j-w/p} \end{aligned}$$

with $\tau (p):=\min \left\{ \frac{\sigma }{p},\ \log \left( 1-\frac{\rho }{p\varepsilon }\right) ^{-1}\right\}$ and $|\!|\!|Y_j|\!|\!|^{w/p}_{u_j}\le \gamma ^{-1}|\!|\!|P_j|\!|\!|^{w/p}_{u_j}$. We check the following bounds

94 $$\begin{aligned} &  2 e\gamma ^{-1}|\!|\!|P_j|\!|\!|^{w/p}_{u_j}\le \frac{1}{6} \end{aligned}$$

95 $$\begin{aligned} &  2 e^{-K\tau (p)}\le \frac{1}{6} \end{aligned}$$

96 $$\begin{aligned} &  2|\!|\!|[ Y_j,\ \sum _{i=0}^{j}\bar{P}_i ]|\!|\!|^{w/p}_{u_j-w/p}\le \frac{1}{6} |\!|\!|P_j|\!|\!|^{w/p}_{u_j} \end{aligned}$$

which will imply (90) with *j* replaced by $j+1$ after dehomogeneizating the weight. As a consequence of (93) (and (90) if $j>1$), we have

$$\begin{aligned} 2 e\gamma ^{-1}|\!|\!|P_j|\!|\!|^{w/p}_{u_j}\le 2 e\gamma ^{-1}|\!|\!|P_1|\!|\!|^{w/p}_{u_1}\le \frac{8}{C^2}\le \frac{1}{6}\qquad C\ge 4\sqrt{3} \end{aligned}$$

so (94) is proved. Moreover, the choice of *p* in (85) guarantees that

$$\begin{aligned} K\tau (p)=K\min \left\{ \frac{\sigma }{p},\ \log \left( 1-\frac{\rho }{p\varepsilon }\right) ^{-1}\right\} \ge \frac{K{\bar{\sigma }}}{p}\ge \log (12) \end{aligned}$$

which gives (95). It remains to prove (96). Using Lemma 4.2 with $Y=\bar{P}_i$, $W=Y_j$, $u_0=u_i$, $u=u_j$, *w* replaced by *w*/*p*, we get

$$\begin{aligned} 2|\!|\!|[ Y_j,\ \sum _{i=0}^{j}\bar{P}_i ]|\!|\!|^{w/p}_{u_j-w/p}\le &  2\sum _{i=0}^{j}|\!|\!|[ Y_j,\ \bar{P}_i ]|\!|\!|^{w/p}_{u_j-w/p}\nonumber \\\le &  2\sum _{i=0}^{j}|\!|\!|P_i|\!|\!|^{w/p}_{u_{j}-w/p}|\!|\!|Y_j|\!|\!|^{2(j-i)w/p+w/p}_{u_j}+|\!|\!|Y_j|\!|\!|^{2(j-i)w/p+w/p}_{u_j-w/p} |\!|\!|P_i|\!|\!|^{w/p}_{u_{i}}\nonumber \\= &  2\sum _{i=0}^{j}\frac{1}{2(j-i)+1}|\!|\!|P_i|\!|\!|^{w/p}_{u_{j}-w/p}|\!|\!|Y_j|\!|\!|^{w/p}_{u_j}+|\!|\!|Y_j|\!|\!|^{w/p}_{u_j-w/p} |\!|\!|P_i|\!|\!|^{w/p}_{u_{i}}\nonumber \\\le &  4 p|\!|\!|Y_j|\!|\!|^{w/p}_{u_j}\sum _{i=0}^{j}\frac{|\!|\!|P_i|\!|\!|^{w}_{u_i}}{2(j-i)+1}\nonumber \\\le &  4p\gamma ^{-1}|\!|\!|P_j|\!|\!|^{w/p}_{u_j}\sum _{i=0}^{j}\frac{|\!|\!|P_i|\!|\!|^{w}_{u_i}}{2(j-i)+1}\nonumber \\= &  c|\!|\!|P_j|\!|\!|^{w/p}_{u_j} \end{aligned}$$

with

$$\begin{aligned} c:= &  4p\gamma ^{-1}\sum _{i=0}^{j}\frac{|\!|\!|P_i|\!|\!|^{w}_{u_i}}{2(j-i)+1}=4p\gamma ^{-1}\frac{|\!|\!|P_0|\!|\!|^{w}_{u_0}}{2j+1}+8p\gamma ^{-1}|\!|\!|P_1|\!|\!|^{w}_{u_1}\\ \le &  4p\gamma ^{-1}\frac{|\!|\!|P_0|\!|\!|^{w}_{u_0}}{2j+1}+32p e \gamma ^{-2}\left( |\!|\!|P_0|\!|\!|^w_{u}\right) ^2\nonumber \\\le &  \frac{8}{eC}+\frac{32}{peC^2}\le \frac{1}{6}\qquad (C\ge 48) \end{aligned}$$

This completes the induction. Choosing now

$$\begin{aligned}p=p_*:=\left[ \frac{K{\bar{\sigma }}}{\log (12)}\right] \qquad j=p_*+1\end{aligned}$$

we obtain

$$\begin{aligned}X_{*}:=X_{p_*+1}=N+G_*+P_{_*}\end{aligned}$$

with $$P_*:=P_{p_*+1}$$ verifying

$$\begin{aligned}|\!|\!|P_{*}|\!|\!|^w_{u-4w}\le \frac{1}{2^{p_*+1}}|\!|\!|P_{0}|\!|\!|^w_{u}\le 2^{- \frac{K{\bar{\sigma }}}{\log (12)} }|\!|\!|P_{0}|\!|\!|^w_{u} \le e^{-K{\bar{\sigma }}/4}|\!|\!|P_{0}|\!|\!|^w_{u}\end{aligned}$$

and $G_*:=\sum _{i=0}^{p_*}\bar{P}_i$ verifying (by (92) and (90))

$$\begin{aligned}|\!|\!|G_*-\bar{P}_0|\!|\!|^w_{u-4w}=\big |\!|\!|\sum _{i=1}^{p_*}\bar{P}_i\big |\!|\!|^w_{u-4w}\le 2 |\!|\!|P_1|\!|\!|^w_{u-4w}\le 8 e \gamma ^{-1}\left( |\!|\!|P_0|\!|\!|^w_{u}\right) ^2.\end{aligned}$$

We finally prove (34). By (31), the transformations $\phi _j$ in (87) verify

$$\begin{aligned} |\phi _{j}-\textrm{id}|_{u_{j-1}-2w_{j-1}}^{w_{j-1}}\le \gamma ^{-1}|\!|\!|P_{j-1}|\!|\!|_{u_{j-1}}^{w_{j-1}},\quad j=1,\ldots ,p_*+1 \end{aligned}$$

Then $$\phi _*:=\phi _1\circ \cdots \circ \phi _{p_*+1}$$

$$\begin{aligned} |\phi _{*}-\textrm{id}|_{u-4w}^{w}\le &  \sum _{j=1}^{p_*+1} |\phi _{j}-\textrm{id}|_{u-4w}^{w}=|\phi _{1}-\textrm{id}|_{u-2w}^{w}+ \sum _{j=2}^{p_*+1} |\phi _{j}-\textrm{id}|_{u-4w}^{w}\nonumber \\= &  |\phi _{1}-\textrm{id}|_{u-2w}^{w}+\frac{1}{p_*} \sum _{j=2}^{p_*+1} |\phi _{j}-\textrm{id}|_{u-4w}^{w_j}\nonumber \\\le &  \gamma ^{-1}|\!|\!|P_{0}|\!|\!|_{u_{0}}^{w_{0}}+\gamma ^{-1}\frac{1}{p_*} \sum _{j=2}^{p_*+1} |\!|\!|P_{j-1}|\!|\!|_{u_{j-1}}^{w_{j-1}}\nonumber \\\le &  \gamma ^{-1}|\!|\!|P_{0}|\!|\!|_{u_{0}}^{w_{0}}+2\gamma ^{-1}\frac{1}{p_*}|\!|\!|P_{1}|\!|\!|_{u_{1}}^{w_{1}}=\gamma ^{-1}|\!|\!|P_{0}|\!|\!|_{u_{0}}^{w_{0}}+2\gamma ^{-1}|\!|\!|P_{1}|\!|\!|_{u_{0}-2w_0}^{w_{0}}\nonumber \\\le &  2\gamma ^{-1}|\!|\!|P_{0}|\!|\!|_{u_{0}}^{w_{0}} \end{aligned}$$

having used (92) in the last step.

## Proof of Proposition 2.1

The eigenvalue–eigenvector equation for the matrix *L*, namely,

$$\begin{aligned} L y=\lambda y\qquad \lambda \in { \mathbb {C}},\ y\in { \mathbb {C}}^4\setminus \{0\} \end{aligned}$$

can be equivalently formulated as the request that the ODE

97 $$\begin{aligned} \dot{x}(t)=L x(t) \end{aligned}$$

has the solution $x(t)=e^{\lambda t}y$. In turn, writing

98 $$\begin{aligned} x=\left( \begin{array}{lll} x_1\\ x_1'\\ x_2\\ x_2' \end{array} \right) ,\quad y=\left( \begin{array}{lll} y_1\\ y_1'\\ y_2\\ y_2' \end{array} \right) \end{aligned}$$

and defining

$$\begin{aligned} \textrm{x}:=\left( \begin{array}{lll} x_1\\ x_2 \end{array} \right) \end{aligned}$$

by multiplying the first and the third equation of (97) by $\epsilon$, $\theta$, respectively, and taking their time–derivative, we obtain the second–order, two–dimensional ODE

99 $$\begin{aligned} T\ddot{\textrm{x}}+B\dot{\textrm{x}}+V\textrm{x}=0, \end{aligned}$$

where

$$\begin{aligned} T:= \left( \begin{array}{cc} \epsilon & 0\\ 0 & \theta \end{array}\right) , \quad B:=\theta \left( \begin{array}{cc} \epsilon & -\epsilon \\ -\epsilon & \delta \end{array}\right) ,\quad V:=2 \left( \begin{array}{cc} c_1\epsilon & 0\\ 0& c_2\theta \end{array}\right) . \end{aligned}$$

Thus, we equivalently look for solutions of (99) of the form

100 $$\begin{aligned} \textrm{x}(t) = e^{\lambda t}\textrm{y},\quad \textrm{with }\ \textrm{y}\in {\mathbb {C}}^2\setminus \{0\} \end{aligned}$$

up to recover the eigenvector *y* in (98) via the relations

$$\begin{aligned} \textrm{y}=\left( \begin{array}{lll} y_1\\ y_2 \end{array} \right) ,\qquad \left( \begin{array}{lll} y'_1\\ y'_2 \end{array} \right) :=\lambda \left( \begin{array}{lll} y_1\\ y_2 \end{array} \right) . \end{aligned}$$

Note that *T*, *B* and *V* are real and symmetric^5^ and their respective minimum, maximum eigenvalues are given by/satisfy

101 $$\begin{aligned} \lambda ^T_-= &  \epsilon ,\quad \lambda ^T_+=\theta \nonumber \\ \lambda ^B_-= &  \frac{\theta }{2}\left( \epsilon +\delta -\sqrt{(\epsilon +\delta )^2-4\epsilon \upupsilon }\right) \ge \frac{\theta \epsilon \upupsilon }{\epsilon +\delta } \nonumber \\ \lambda ^B_+= &  \frac{\theta }{2}\left( \epsilon +\delta +\sqrt{(\epsilon +\delta )^2-4\epsilon \upupsilon }\right) \le \theta (\epsilon +\delta ) \nonumber \\ \lambda ^V_-= &  2\min \{c_1\epsilon ,c_2\theta \}\ge 2\epsilon \min \{c_1,c_2\},\nonumber \\\lambda ^V_+= &  2\max \{c_1\epsilon ,c_2\theta \}\le 2\theta \max \{c_1,c_2\}. \end{aligned}$$

Replacing (100) into (99) and taking the Hermitian inner product (here denoted as $(\cdot , \cdot )$) with ${\textrm{y} }$ leads to relation:

$$\begin{aligned} \lambda ^2(\textrm{y},T\textrm{y} )+\lambda (\textrm{y},B\textrm{y} )+(\textrm{y},V\textrm{y} )=0. \end{aligned}$$

We solve for $\lambda$:

102 $$\begin{aligned} \lambda = -\frac{(\textrm{y},B\textrm{y} )}{2(\textrm{y},T\textrm{y} )} \pm \textrm{i}\frac{\sqrt{ 4(\textrm{y},T\textrm{y} )(\textrm{y},V\textrm{y} )-(\textrm{y},B\textrm{y} )^2 }}{2(\textrm{y},T\textrm{y} )}. \end{aligned}$$

As Equation (102) does not change multiplying $\textrm{y}$ by an arbitrary $c\in {\mathbb {C}}\setminus \{0\}$, we do not loose generality if we assume $(\textrm{y},\textrm{y} )=1$. Under such assumption, by the min-max principle, the expression under the square root is bounded below by

103 $$\begin{aligned} 4 \lambda ^T_{-} \lambda ^{V}_{-}-(\lambda ^B_{+})^2 &  \ge 8\epsilon ^2\min \{c_1,c_2\}-\theta ^2(\epsilon +\delta )^2>8\epsilon ^2\min \{c_1,c_2\}-9\theta ^2\epsilon ^2 \nonumber \\  &  \ge 4\epsilon ^2\min \{c_1,c_2\} \end{aligned}$$

and above by

104 $$\begin{aligned} {4 \lambda ^T_{+} \lambda ^{V}_{+}\le 8\theta ^2\max \{c_1,c_2\}} \end{aligned}$$

having used (5), (10) and (101). Equations (102) and (103) show that the eigenvalues of *L* come in complex conjugated couples with non–vanishing imaginary part. As we have assumed that the resolvent of the characteristic polynomial of *L* does not vanish, *L* has two distinct such couples. Moreover, again from (5), (101), (102) and (104), we have

$$\begin{aligned} {\, \mathrm Re\, }\lambda= &  -\frac{(\textrm{y},B\textrm{y} )}{2(\textrm{y},T\textrm{y} )}\in \left[ -\frac{\lambda _+^B}{2\lambda ^T_-},\ -\frac{\lambda ^B_-}{2\lambda ^T_+} \right] \subset \left[ -\frac{\theta (\epsilon +\delta )}{2\epsilon },\ -\frac{\epsilon \upupsilon }{{2}(\epsilon +\delta )}\right] \subset \left[ -\frac{3}{2}\theta ,-\frac{\upupsilon }{{6}}\right] \nonumber \\ {|{\, \mathrm Im\, }\lambda |}= &  {\frac{\sqrt{ 4(\textrm{y},T\textrm{y} )(\textrm{y},V\textrm{y} )-(\textrm{y},B\textrm{y} )^2 }}{2(\textrm{y},T\textrm{y} )} \subset \left[ \frac{\epsilon }{\theta }\sqrt{\min \{c_1, c_2\}},\ \frac{\theta }{\epsilon }\sqrt{2\max \{c_1, c_2\}}\right] } \end{aligned}$$

which proves (12). $$\square $$

### Remark 5.1

The procedure here used to prove Proposition 2.1 is considerably simpler than a strategy based on the analysis of the characteristic polynomial of *L*, which is given by $P(\lambda )=(\lambda ^2+\theta \lambda +2c_1)(\lambda ^2+\delta \lambda +2c_2)-\theta \epsilon \lambda ^2$. Remark that the same argument may be applied whenever one needs to infer algebraic properties of the eigenvalues of any $n\times n$ matrix *L* whose ODE (97) may be put in the form (99), with *T*, *B* and *V* Hermitian.

## Data Availability

This work has no associated data.

## Proof of Proposition 4.1

In general, a diffeomorphism $x=\Phi (y)$ transforms the Equation (25) to $\dot{y}=Z(y)$, where

$$\begin{aligned}Z(y)=J(y)^{-1}X\big (\Phi (y)\big )\end{aligned}$$

with *J*(*y*) being the Jacobian matrix of the transformation, i.e.,

$$\begin{aligned}J(y)_{hk}=\partial _{y_k}\Phi _h(y),\quad \textrm{if}\quad \Phi =(\Phi _1, \ldots , \Phi _n).\end{aligned}$$

Applying this to the flow $\Phi _\tau ^Y$ in Definition 4.1, we obtain that the new vector–field is

$$\begin{aligned}Z_\tau (y):=J_\tau ^Y(y)^{-1}X\big (\Phi ^Y_\tau (y)\big )\quad \textrm{with} \quad (J_\tau ^Y(y))_{hk}:=\partial _{y_k}\big (\Phi ^Y_\tau (y)\big )_h.\end{aligned}$$

We stress that the thesis of Proposition 4.1 is an immediate consequence of the following identity

105 $$\begin{aligned} \frac{d^k}{dt^k} Z_t(y)=J_{t}^Y(y)^{-1}{\mathcal {L}}^k_Y X\big (\Phi ^Y_{t } (y)\big )\quad \forall \ 0\le t\le \tau \end{aligned}$$

which we are going to prove. Indeed, (105) implies

$$\begin{aligned}\frac{d^k}{dt^k} Z_t(y)\Big |_{t=0}={\mathcal {L}}^k_Y X\big (y\big )\end{aligned}$$

which gives

$$\begin{aligned}Z(y)=Z_\tau (y)=\sum _{k=0}^\infty \frac{\tau ^k}{k!}\frac{d^k}{dt^k} Z_t(y)\Big |_{t=0}=\sum _{k=0}^\infty \frac{\tau ^k}{k!}{\mathcal {L}}^k_Y X\big (y\big )=e^{\tau {\mathcal {L}}_Y}X(y).\end{aligned}$$

Taking $\tau =1$ will give the proof of Proposition 4.1.

Let us then prove (105). We use the expansion

106 $$\begin{aligned} \Phi ^Y_t (y)=\Phi ^Y_{t _0} (y)+Y\big (\Phi ^Y_{t _0} (y)\big )(t -t _0)+o(t -t _0)\end{aligned}$$

and

107 $$\begin{aligned} J_t ^Y(y)=\Big ({{\mathbb {I}}}+J_Y\big (\Phi ^Y_{t _0} (y)\big )(t -t _0)\Big )J_{t _0}^Y(y)+o(t -t _0)\qquad J_Y(z)_{hk}=\partial _{z_k}Y_h(z).\end{aligned}$$

Equation (107) gives

108 $$\begin{aligned} (J_t ^Y(\eta ))^{-1}=(J_{t _0}^Y(y))^{-1}\Big ({{\mathbb {I}}}-J_Y\big (\Phi ^Y_{t _0} (y)\big )(t -t _0)\Big )+o(t -t _0).\end{aligned}$$

While (106) gives

109 $$\begin{aligned} X\big (\Phi ^Y_t (y)\big )= &  X\big (\Phi ^Y_{t _0} (y)+Y\big (\Phi ^Y_{t _0} (y)\big )(t -t _0)+o(t -t _0)\big )\nonumber \\= &  X\big (\Phi ^Y_{t _0} (y)\big )+J_X\big (\Phi ^Y_{t _0} (y)\big ) Y\big (\Phi ^Y_{t _0} (y)\big )(t -t _0)+o(t -t _0) \end{aligned}$$

Collecting (108) and (109), we then find

$$\begin{aligned} Z_t (y)= &  J_t ^Y(y)^{-1}X\big (\Phi ^Y_t (y)\big )\nonumber \\= &  J_{t _0}^Y(y)^{-1} \nonumber \\ &  \Big ({{\mathbb {I}}}-J_Y\big (\Phi ^Y_{t _0} (y)\big )(t -t _0)+o(t -t _0)\Big )\Big (X\big (\Phi ^Y_{t _0} (y)\big )+J_X\big (\Phi ^Y_{t _0} (y)\big ) Y\big (\Phi ^Y_{t _0} (y)\big )(t -t _0)\Big )\nonumber \\ &  +o(t -t _0)\nonumber \\= &  J_{t _0}^Y(y)^{-1}X\big (\Phi ^Y_{t _0} (y)\big )\nonumber \\ &  +J_{t _0}^Y(y)^{-1}\Big (J_X\big (\Phi ^Y_{t _0} (y)\big ) Y\big (\Phi ^Y_{t _0} (y)\big )-J_Y\big (\Phi ^Y_{t _0} (y)\big ) X\big (\Phi ^Y_{t _0} (y)\big )\Big )(t -t _0)+o(t -t _0) \end{aligned}$$

This expansion shows that

$$\begin{aligned}\frac{d}{dt } Z_t (y) =\frac{d}{dt }\Big ( J_{t }^Y(y)^{-1}X \big (\Phi ^Y_{t } (y)\big )\Big ) =J_{t }^Y(y)^{-1}{\mathcal {L}}_Y X\big (\Phi ^Y_{t } (y)\big ) \end{aligned}$$

By iteration, we have (105). $\square$

## Footnotes

1 *arXiv:1710.02689.*

2 We sketchily justify the formulae in (3), leaving the details to the interested reader. The gravitational potential generated by the shell–core system has an expansion in powers of the distance $\rho$ between its center and the attracting mass given by (see (Pinzari et al., 2024, Eq. (2)))

4 $$\begin{aligned} {{\mathcal {V}}}+{{\mathcal {V}}}'=-\frac{GM}{\rho }\Big \{ m+\frac{1}{4\rho ^2}\Big [A+B+3 (B-A)\cos 2\nu +A'+B'+3(B'-A')\cos 2\varphi \Big ]+\ldots \Big \} \end{aligned}$$

where *G* is the gravity constant, *m*, *M* are the masses of the rotating and the attracting body, $\rho$ is the distance among them, *A*, *B*, $A'$, $B'$ are the moments of inertia, $\varphi$, $\nu$ are angles describing rotations of shell and core, respectively, and finally the dots convey terms which begin with power $\rho ^{-3}$. The lowest order term in (4), $-\frac{GMm}{\rho }$, is kicked out, because plays the rôle of orbital potential for the variable $\rho$ (see the term $E_k$ in (Pinzari et al. 2024, Eq. (4))). The remaining part is $O(r^2/a^3)$ (because *A*, *B*, $A'$, $B'=O(r^2)$ while $\rho =O(a)$) and is eventually split into the sum of its $\ell$–average, which here we denote as $\overline{{\mathcal {V}}}+\overline{{\mathcal {V}}}'$, plus a zero–average part $\widetilde{{\mathcal {V}}}+\widetilde{\mathcal{V}}'$. Proceeding similarly as in (Pinzari et al., 2024, Eq. (7)-(13)) one can show that the lowest order terms of $\overline{{\mathcal {V}}}+\overline{{\mathcal {V}}}'$ are precisely the terms with $\cos 2\gamma$, $\cos 2\eta$ in (3). Higher order terms in the expansion of $\overline{{\mathcal {V}}}+\overline{{\mathcal {V}}}'$ and terms coming from the dots in (4) correspond to the function $\widehat{{\mathcal {V}}}$ in (3) and are readily seen to be $O(r^3/a^4)$.

3 As discussed in Pinzari et al. (2024), the terms $a_k(e)$ come from the mean anomaly–eccentricity expansion of $\left( \frac{a}{\rho }\right) ^3$ which, as known from the literature, has the form

$$\begin{aligned}\left( \frac{a}{\rho }\right) ^3=\sum _{k=0}^{\infty } a_k(e)e^k \cos k\ell \end{aligned}$$

with $$a_k(0)=1$$.

4 Compare Eq. (25) in Pinzari et al. (2024) and the comment below.

5 The multiplication by $\epsilon$, $\theta$ allowed to have the matrix *B* symmetric, keeping *T* and *V* (diagonal, hence) symmetric.
